# Synaptic Integration of Thalamic and Limbic Inputs in Rodent Gustatory Cortex

**DOI:** 10.1523/ENEURO.0199-19.2019

**Published:** 2020-02-13

**Authors:** M. E. Stone, A. Fontanini, A. Maffei

**Affiliations:** Department of Neurobiology and Behavior, State University of New York-Stony Brook, Stony Brook, NY 11794

**Keywords:** amygdalocortical, excitation, gustatory cortex, inhibition, integration, thalamocortical

## Abstract

Neurons in the gustatory cortex (GC) process multiple aspects of a tasting experience, encoding not only the physiochemical identity of tastes, but also their anticipation and hedonic value. Information pertaining to these stimulus features is relayed to GC via the gustatory thalamus (VPMpc) and basolateral amygdala (BLA). It is not known whether these inputs drive separate groups of neurons, thus activating separate channels of information, or are integrated by neurons that receive both afferents. Here, we used anterograde labeling and *in vivo* intracellular recordings in anesthetized rats to assess the potential convergence of BLA and VPMpc inputs in GC, and to investigate the dynamics of integration of these inputs. We report substantial anatomic overlap of BLA and VPMpc axonal fields across GC, and identify a population of GC neurons receiving converging BLA and VPMpc inputs. Our data show that BLA modulates the gain of VPMpc-evoked responses in a time-dependent fashion and that this modulation is dependent on the recruitment of synaptic inhibition by both BLA and VPMpc. Our results suggest that BLA shapes cortical processing of thalamic inputs by dynamically gating the excitatory/inhibitory balance of the GC circuit.

## Significance Statement

The gustatory cortex (GC) is ideal for studying sensory and limbic signals to primary sensory cortices. GC encodes the chemosensory and affective components of taste by integrating inputs from thalamus and basolateral amygdala (BLA). Classic studies postulated a separation of sensory and limbic channels. However, reports of GC neurons encoding taste, palatability, and expectation in their dynamic firing modulations challenge this view. Here, we show the synaptic basis of the convergence and integration of thalamic and amygdalar inputs onto single GC neurons. Our data reveal a population of GC neurons receiving monosynaptic drive from both regions and show that amygdalar inputs can gate the processing of thalamic signals. Altogether, our data demonstrate that GC integrates, rather than segregates, these channels.

## Introduction

Sensory perception depends on the integration of sensory and affective features of stimuli, yet how these properties are encoded and processed by cortical neurons is not well understood. The primary gustatory cortex (GC) provides a unique model for investigating this question, as GC neurons in alert animals multiplex sensory, affective, and associative signals (Maffei et al., 2012; Vincis and Fontanini, 2016b). Indeed, GC neurons encode information about the physiochemical identity and hedonic value of tastants, along with anticipatory activity, in the temporal dynamics of their spiking responses (Yamamoto et al., 1981, 1984a,b; Katz et al., 2001; Pritchard and Norgren, 2004; Spector and Travers, 2005; Fontanini and Katz, 2006; Yokota et al., 2007; Grossman et al., 2008; Piette et al., 2012; Sadacca et al., 2012, 2016; Samuelsen et al., 2012, 2013; Jezzini et al., 2013; Maier and Katz, 2013; Gardner and Fontanini, 2014; Moran and Katz, 2014; Vincis and Fontanini, 2016a). Experimental evidence indicates that chemosensory information is relayed to GC primarily via thalamocortical afferents from the parvicellular division of the ventroposteromedial nucleus (VPMpc; Saper, 1982; Kosar et al., 1986; Pritchard et al., 1989; Allen et al., 1991; Nakashima et al., 2000; Verhagen et al., 2003; Samuelsen et al., 2013; Liu and Fontanini, 2015), while affective dimensions, including palatability and expectations about incoming stimuli, depend on a limbic contribution from the basolateral amygdala (BLA; Saper, 1982; Yamamoto et al., 1984a; Allen et al., 1991; Grossman et al., 2008; Fontanini et al., 2009; Piette et al., 2012; Samuelsen et al., 2012).

The existing literature does not provide clear evidence about how thalamic and limbic channels of information are jointly processed by GC. Per one model, they could independently reach different GC neurons and either be processed independently, or be integrated multi-synaptically by intracortical circuits (Shi and Cassell, 1998; Sewards and Sewards, 2001). Another possibility is that both inputs could converge onto a group of GC neurons via direct monosynaptic projections and be integrated in the temporal dynamics of the response arising from the activation of both inputs. A third model could depend on a hybrid organization in which limbic and thalamic inputs are integrated directly by a group of neurons receiving converging input and indirectly via intracortical connectivity.

Tracing studies report that VPMpc projections primarily terminate in the granular and dysgranular subdivision of GC while BLA projections are mostly observed in the dysgranular and agranular subdivisions (Allen et al., 1991; Nakashima et al., 2000). These data were interpreted as evidence for the segregation of the limbic and thalamic channels of information to GC. However, a detailed anatomic and functional analysis of VPMpc and BLA terminal fields in the same GC preparation has not been performed. Recordings in behaving rodents have shown that the same GC neuron can encode taste quality, expectation, and palatability. This work has been interpreted as evidence for the integration of information onto GC neurons, although the results remain agnostic regarding whether the convergence of information depends on intracortical interactions or direct projections onto a subset of GC neurons (Fontanini and Katz, 2006; Samuelsen et al., 2012; Gardner and Fontanini, 2014).

Here, using double anterograde tracing, confocal microscopy and intracellular recordings in anesthetized rats, we show that there is significant overlap of VPMpc and BLA axonal fields in GC. The evidence for anatomic overlap of VPMpc and BLA inputs is confirmed and expanded by analysis of evoked responses recorded intracellularly in anesthetized rats, revealing a channel of convergence for thalamic and limbic afferents in GC. Finally, in view of inactivation studies demonstrating that silencing VPMpc completely abolishes GC taste responses, while inactivation of BLA modulates taste responses but does not abolish them (Samuelsen et al., 2013), we chose to assess a potential modulatory role of BLA inputs on VPMpc responses. We show that GC neurons’ responsiveness to VPMpc stimulation can indeed be modulated by BLA stimulation in a time-dependent manner. Two groups of neurons were identified based on the sign (facilitation or suppression) of modulation of VPMpc responses by BLA bursts. These groups differed significantly in the amplitude of the polysynaptic inhibitory (hyperpolarizing) component evoked by single BLA and VPMpc stimuli, revealing that the BLA modulation of the gain of VPMpc inputs depends both on the relative timing of the two stimuli and on their ability to recruit inhibitory circuits in GC.

To our knowledge, this study is the first report on the convergence of thalamic and amygdalar inputs onto GC neurons and their dynamics of integration. These results provide a circuit model for how, in a primary sensory area, the coding of sensory stimuli becomes enriched with information about their affective dimensions.

## Materials and Methods

### Subjects

Female Long Evans rats (275–330 g; *n* = 22) were purchased from Charles River, housed individually in a vivarium, and maintained on a 12/12 h light/dark cycle with *ad libitum* access to standard chow and water. Female rats accumulate less body fat than age matched males, facilitating the achievement and maintenance of stable surgical anesthesia throughout the experimental procedures. All study methods were performed following state, federal and university regulations, and were approved by the Institutional Animal Care and Use Committee.

### Fluorescent anterograde labeling and image analysis

Subjects (*n* = 3) were surgically prepared for virus injection for anterograde labeling as follows: Anesthesia was induced with an intraperitoneal injection of ketamine/xylazine/acepromazine cocktail (KXA; 100, 5.2, and 1 mg/kg, respectively). Anesthesia depth was monitored via the hind limb withdrawal reflex and respiratory rate. Supplemental doses of KXA, 30% of induction dose, were administered as necessary to maintain a surgical plane of anesthesia. 37 ± 1°C body temperature was maintained with a heating pad connected to a rectal thermometer (FHC Inc). Rats were mounted on a stereotaxic apparatus (Narishige), bupivacaine was applied to pressure points and incision sites, and the scalp was incised. Bregma and λ were leveled, burr holes were drilled, and the dura overlying the gustatory thalamus (VPMpc) and BLA was opened. The following stereotaxic coordinates from bregma were used to target the injections: VPMpc, −3.6 mm posterior, −1.0 mm lateral, and −6.5 mm ventral from the pia; BLA, −3.0 mm posterior, −5.0 mm lateral, and −7.2 mm ventral from the pia (Paxinos and Watson, 2007). The exposed cortex was covered in 0.9% saline to prevent drying.

Rats were injected with a construct of the vector adeno-associated virus serotype 9 (AAV9) and the gene expressing either green fluorescent protein (GFP; AAV9.hSyn.eGFP.WPRE.bGH, UPENN vector core) or red fluorescent protein (RFP; AAV9.hSyn.TurboRFP.WPRE.rBG, UPENN vector core) into bilateral VPMpc and BLA, with the GFP and RFP injection location counterbalanced within the group. The AAV9 subtype was chosen for its preferred anterograde transport (Aschauer et al., 2013). AAV9 is transported anterogradely, as assessed in our previous studies in which the same viral construct was used (Wang et al., 2013, 2019; Kloc and Maffei, 2014; Haley et al., 2016). This property was further confirmed by the lack of retrogradely labeled somata in GC in all preparations used for this study. AAV9 constructs were pressure-injected with a Hamilton syringe at an approximate rate of 50 μl every 20 s. Total injection volumes of 300 nl in VPMpc and 500 nl in BLA were delivered (50 × 10^12^ viral particles/nl). This procedure resulted in the expression of GFP or RFP localized to the injection site after a 14- to 21-d incubation period. Following incubation, rats were deeply anesthetized with KXA and transcardially perfused with 0.9% saline followed by cold 4% paraformaldehyde in PBS. Brains were dissected, postfixed in 4% paraformaldehyde overnight, and then stored in 30% sucrose solution in 0.1 M PB for cryoprotection; 100-μm coronal sections were prepared with a cryostat and collected in 0.1 M PBS. Sections for confirming BLA and VPMpc injection sites were incubated on a shaker for 30 min in a 1:5000 solution of Hoechst (Life Technologies) in 0.1% Triton X-100. Sections were rinsed twice with 0.1 M PBS for 10 min and twice again with 0.1 M PB for 10 min before being mounted (Fluoromount G; Southern Biotechnology) and coverslipped. Confocal images of VPMpc and BLA injection sites were taken with 15–20× objectives using an Olympus FV-1000 with IX-81 inverted microscope controlled by FV10-ASW software (Olympus Imaging America Inc.). Sections for GC imaging were incubated on a shaker for 60 min 0.1% Triton X-100 in PBS (Life Technologies) followed by a 30 min incubation in a 1:200 solution of blue fluorescent Nissl (Life Technologies) in 0.1 M PBS. Sections were rinsed twice with 0.1 M PBS for 10 min and twice again with 0.1 M PB for 10 min before being mounted (Fluoromount G; Southern Biotechnology), and coverslipped. Confocal images of VPMpc and BLA axonal fields in GC were taken under 20–60× magnification.

Confocal images were viewed and analyzed using ImageJ software (National Institutes of Health). The intensity of the GFP and RFP signal was used to quantify the density of the BLA and VPMpc projections in regions of interest (ROIs) outlined in GC. Two linear ROIs were drawn orthogonal to the GC subdivisions extending 2 mm from piriform cortex (Pir.) toward somatosensory cortex (Som.). One ROI was positioned in the superficial layers (border between layers 2/3 and 4, ROI A) and one ROI was positioned in the deep layers (layers 5/6; ROI B). For the ROI analysis, thee 100-μm GC slices were used from each of the three rats for a total of nine slices. ROIs (*n* = 18) were drawn in the same hemisphere across the three slices for each rat to facilitate comparison. RFP and GFP fluorescence intensity was measured along each ROI in each slice and normalized to the maximum signal. The normalized data were pooled across rats, averaged in 50-μm bins, and the mean ± SD was plotted against dorsoventral (DV) distance through the subdivisions.

### *In vivo* intracellular recording

Rats (*n* = 19) were anesthetized with an intraperitoneal injection of 40 mg/kg pentobarbital and 0.6 g/kg urethane (Yokota et al., 2007; Stone et al., 2011). Anesthesia depth was monitored via the hind limb withdrawal reflex, respiratory rate, and EEG. Supplemental doses of urethane, 20–30% of the induction dose, were administered as necessary for maintenance of a surgical plane of anesthesia. Recordings were performed several hours after induction and we expect the initial dose of pentobarbital had worn off by that time. Bupivacaine was applied to all incisions and pressure points. Rats were tracheotomized to facilitate breathing, and 37 ± 1°C body temperature was maintained with a heating pad connected to a rectal thermometer (FHC Inc). Following these procedures, rats were mounted on a stereotaxic apparatus (Narishige) and the scalp was incised. Bregma and λ were leveled, burr holes were drilled dorsally above the gustatory portion of the insular cortex (GC), gustatory thalamus (VPMpc), and, in a subset of *n* = 10/19 rats, BLA. A dorsal approach was adopted, relying on the following coordinates from bregma as per the stereotaxic atlas: GC, +1.5 mm anterior and −5.0 mm lateral; VPMpc, −3.6 mm posterior and −1.0 mm lateral; BLA, −3.0 mm posterior and −5.0 mm lateral (Paxinos and Watson, 2007). The dura was removed for each craniotomy and the exposed cortex was covered in mineral oil to prevent drying. Two additional small burr holes were drilled over parietal cortex and cerebellum, and stainless-steel set screws were inserted to facilitate EEG recording of anesthesia depth. A tungsten bipolar stimulating electrode (0.1 mΩ, 250-μm tip separation; MicroProbes for Life Science) was lowered into VPMpc (final DV coordinate determined as described below). In a subset of *n* = 10/19 rats, a concentric bipolar stimulating electrode (MicroProbes for Life Science) was lowered into BLA, 7.2 mm ventral from the pia.

For each preparation included in the analysis, the position of the stimulating electrode in VPMpc was established by mapping thalamic responses to a taste stimulus in each animal. The final DV depth of the VPMpc stimulating electrode from the pial surface was guided by single-unit and multi-unit activity evoked by the application of cold 0.3 M NaCl solution to the oral cavity. This taste solution was chosen because it has been shown to be the strongest stimulus for evoking VPMpc activity (Verhagen et al., 2003). NaCl solution and a distilled water rinse were pressure-injected directly into the mouth via polyimide tubing connected to solenoid valves controlled by a computer via a valve driver (model CDET-PH-08, Optimal Engineering Systems). A pilot study using methylene blue showed that our taste delivery system bathes the entire oral cavity with taste solution, including the anterior and posterior tongue, palate, and epiglottis (data not shown). Once single-unit or multi-unit spiking was detected (model 1800 AC amplifier, AM Systems), 10 s of background activity was collected. Then, cold 0.3 M NaCl solution was delivered for 3 s. Ten seconds after the offset of NaCl, a 3-s-long dH2O rinse was delivered. Changes in single-unit or multi-unit firing in response to NaCl were observed online and recorded for offline analysis. The electrode was lowered to the depth of the strongest evoked response (DV range: −6.0 to −6.8 mm).

Intracellular recordings with sharp electrodes were obtained as described previously (Fontanini et al., 2003; Stone et al., 2011). Current clamp recordings were performed in bridge-balance mode using an Axon Multiclamp 700b amplifier (Axon Instruments). Borosilicate capillaries (OD 1.0 mm) were pulled with a Flaming-Brown puller (P97; Sutter Instruments) and filled with 3 M potassium acetate solution containing 2% biocytin (Anaspec) for *post hoc* identification of neurons. *In situ* impedances ranging 35–85 MΩ were measured. Hyperpolarizing current steps (−500 pA, 500 ms, 0.5 Hz) were passed through the electrode while it was slowly lowered ventrally into GC with a hydraulic micromanipulator (Narishige). Sharp electrodes penetrated the cortex in a search area of 1.5 ± 0.5 mm antero-posterior (AP) and 5.0 ± 0.5 mm medio-lateral (ML) from bregma within the craniotomy. The depth of the electrode from the cortical surface was monitored continuously. GC cells in this study were recorded from a DV depth range of −4 to −5.6 mm, consistent with the DV extent of the taste-responsive region of insular cortex (Yamamoto et al., 1984b). Only recordings from GC neurons showing action potentials of at least 50 mV and resting potentials at −60 mV or lower were used for data analysis. Stable recordings typically lasted >60 min in our preparation.

Current clamp intracellular recordings were acquired at 20 kHz with a Digidata 1440A board (Axon Instruments) connected to a computer running Clampex 10 acquisition software (Axon Instruments). Data collection began once the cell had stabilized, typically within 5 min after impalement. Hyperpolarizing current (0.5–2 nA) was injected to stabilize the membrane potential of the cell and this current was discontinued once the membrane reached a stable state.

Baseline activity was recorded for 1 min. The neuron’s firing frequency was calculated as the number of spontaneous action potentials occurring during the baseline trace. Membrane resting potential was calculated from the downstate of the baseline trace, with spontaneous postsynaptic potentials (PSPs) and action potentials excluded. Following baseline recording, steps of DC current (range: −1000 to 1500 pA, 1.5 s, 0.2 Hz) were injected. The input resistance of the cell was calculated from the linear portion of the membrane potential response to hyperpolarizing current steps. Firing rates to depolarizing current steps were plotted as a frequency to current (f/I) curve. Only cells with firing properties consistent with regular spiking pyramidal cells were included in the analysis. All recorded potentials were corrected offline for the liquid junction potential.

GC neurons were tested for responsiveness to electrical VPMpc stimulation and, in rats which had stimulating electrodes in BLA, BLA stimulation. Stimuli were 0.1-ms biphasic pulses delivered to the bipolar, in the case of VPMpc, and concentric, in the case of BLA, stimulating electrodes via two stimulus isolation units (World Precision Instruments) controlled by a Master-8 (A.M.P.I.). Stimulation intensity was adjusted for all cells to evoke the largest subthreshold PSP (intensity range: 0.1–2.25 mA, VPMpc, *n* = 26 cells; 0.2–2.5 mA, BLA, *n* = 13 cells).

### Analysis of electrophysiological data

Data included in the analysis of convergent connectivity were obtained from 30 neurons showing responsiveness to both BLA and VPMpc. Of these neurons, 26 putative pyramidal neurons in GC of 19 rats were sufficiently long to run the tests of connectivity as well as the tests to assess the modulation of VPMpc responses by BLA. Subpopulations of cells, as specified in Results, were tested with different protocols, due to the constraints of limited recording time per impalement. PSP onset latency was measured as the time from stimulus onset to evoked PSP onset. PSP peak amplitude was measured as the greatest membrane potential deflection from PSP onset. PSP time-to-peak was measured as the time from stimulus onset to peak PSP amplitude. Rarely, we recorded neurons with either all-or-none spiking responses that collided with preceding action potentials and/or very short (<2 ms) response latencies to VPMpc stimulation consistent with possible antidromic activation. These recordings were terminated, and any data already collected was not analyzed.

The voltage dependency of the early and late synaptic components of the evoked VPMpc-PSP was investigated by using a fixed stimulation intensity that evoked the largest subthreshold PSP (as described above) and manipulating the holding potential of the cell with current injections (*n* = 10 cells tested). Five to eight sweeps at each holding potential were averaged and the PSP amplitudes for the average traces were measured early (3–5 ms post-PSP onset) and late (35 ms post-PSP onset) in the evoked response (Moore and Nelson, 1998; Stone et al., 2011). Amplitude values were normalized for each cell and the normalized values were plotted against the holding potential. The reversal potentials of the components of the PSP were determined by following the linear regression to the *x-intercept*.

A subset of cells was tested for responses to both BLA and VPMpc stimulation and data are presented for a total of 13 GC neurons responding to both inputs with monosynaptic delays. In one group that responded with PSPs to both BLA and VPMpc stimuli (*n* = 5), a single BLA stimulus (sBLA) was followed at varying latencies by a single VPMpc stimulus (sBLA+sVPMpc). Latencies ranged from 0 to 200 ms. For each interstimulus latency, five trials were recorded. The peak amplitude of the evoked PSP to sBLA+sVPMpc was measured as the largest membrane potential deflection following PSP onset. The amplitude was then normalized to that of the evoked PSP to VPMpc stimulation recorded at baseline (sVPMpc). For the purposes of analysis, the latency between the BLA stimulus and VPMpc stimulus was corrected by the difference between the onset latencies of the two PSPs recorded independently before the pairing (Fig. 5*D*, effective interstimulus latency).

Another group of neurons (*n* = 8) was tested with the following stimulation protocol. A single VPMpc stimulus (sVPMpc) was followed 500–2000 ms later by a 500-ms burst of BLA stimulation at 20 Hz (bBLA) paired with a second VPMpc stimulus (bBLA+sVPMpc). The second VPMpc stimulus was delivered 50, 250, or 500 ms post-BLA burst. For each post-burst latency, 16 trials were recorded, peak PSP amplitudes were measured, and mean ± SEM was calculated. The 20-Hz frequency for the BLA train was chosen to mimic the typical response pattern of BLA neurons in response to auditory cues predicting the availability of tastes in extracellular recordings of awake, behaving rats (Samuelsen et al., 2012). The amplitude of the PSP evoked by the VPMpc stimulus following bBLA+sVPMpc pairing was measured as the greatest membrane potential deflection following PSP onset and normalized to the amplitude of the PSP evoked by VPMpc stimulation alone. bBLA+sVPMpc-PSP amplitude was also compared with the sVPMpc-PSP amplitude evoked at comparable holding membrane potential to verify that the effect of the bBLA+sVPMpc pairing on VPMpc-PSPs did not depend solely on a shift in membrane potential. The coefficient of variation was calculated as the mean divided by standard deviation of the peak PSP amplitudes to both VPMpc stimuli for each cell tested. Onset latency was measured as the time from stimulus onset to the evoked PSP onset. Time-to-peak was measured as the time from stimulus onset to peak PSP amplitude, and the time from PSP onset to peak PSP amplitude, as specified in Results. Both onset latency and time-to-peak were measured in each of 16 trials and then averaged for each condition and cell. Rise time and slope of the evoked VPMpc-PSP were measured from 10–90% peak amplitude using the average of 16 traces for each condition in each cell.

### Sharp electrode recording depth analysis

Throughout each penetration of the sharp electrode, and at the time of each recording of an impaled cell, the depth of the electrode’s tip from the pia was monitored. Because of the low yield of recovery of biocytin-stained cells from which complete datasets were collected, this depth measurement was our best proxy for neurons’ locations in GC. The neurons contributing to the data in this study (*n* = 26) were pooled with those from a previous study on GC responses to electrical stimulation of BLA (*n* = 27; Stone et al., 2011) and with cells that were tested for responsiveness but were not included in either dataset due to either poor recording quality or losing the recording before a useable dataset could be collected (*n* = 50). This pooling yielded a sample within which 42 cells were responsive to VPMpc stimulation and 61 cells were responsive to BLA stimulation. Of these neurons, 30 were responsive to both stimuli. Recording depths were compared across these three groups with frequency histograms. The VPMpc-responding and BLA-responding populations were compared by plotting the normalized cumulative sum of their frequency distributions and statistical significance was assessed with the Kolmogorov–Smirnov (KS) test.

### Histology

Recorded GC neurons were filled with biocytin with depolarizing current pulses (500–1000 pA, 300 ms, 1.5 Hz; 10–30 min in duration) whenever the recording length allowed and before abruptly pulling the sharp electrode out of the cell. At the end of the recording session, rats were deeply anesthetized with pentobarbital. For *post hoc* identification of the tips of the stimulating electrodes in VPMpc and BLA, tissue was lesioned by applying DC current (0.2 mA, 20 s). Rats were perfused transcardially with 0.9% saline followed by cold 4% paraformaldehyde in PBS. Brains were dissected and postfixed in cold 4% paraformaldehyde for 2 h before being moved to 30% sucrose solution in 0.1 M PB for cryoprotection. Coronal sections were sliced on a cryostat. VPMpc and BLA lesions were visualized in 60-μm sections stained with cresyl violet according to standard techniques. To identify biocytin-filled GC pyramidal cells, 100-μm sections of GC were stained with DAB and counterstained with cresyl violet according to standard techniques.

## Results

### Anatomical overlap of thalamocortical and amygdalocortical axon terminals in GC

**Figure 1.**
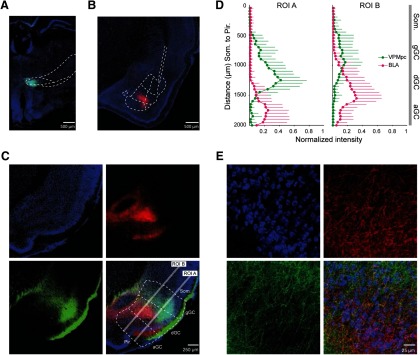
Overlap of thalamocortical and amygdalocortical axonal fields in GC. ***A***, Representative injection site of AAV9-GFP in VPMpc. Coronal section imaged at 20× magnification showing GFP-filled neurons (green) and Hoechst counterstain (blue). ***B***, Representative injection site of AAV9-RFP in BLA. Coronal section at 15× magnification showing RFP-filled neurons (red) and Hoechst counterstain (blue). ***C***, Coronal section of GC at 40× magnification showing GFP-labeled axons from VPMpc (green; bottom left), RFP-labeled axons from BLA (red; top right), counterstain with fluorescent Nissl (cyan; top left), and merge (bottom right). White lines indicate 2-mm linear ROIs used to measure fluorescence intensity dorsoventrally through GC from somatosensory to Pir.; ROI A sampled superficial GC layers (2/3 and 4) while ROI B sampled deep layers (5 and 6). gGC, granular GC; dGC, dysgranular GC; aGC, agranular GC. ***D***, Mean ± SD normalized fluorescence intensity versus DV depth measured in two ROIs, the superficial (ROI A) and deep (ROI B) cortical layers (*n* = 9; 3 slices from each of three rats). While VPMpc fluorescence (green) intensity was greater in the dorsal subdivisions (granular/dysgranular) and BLA fluorescence intensity (red) was greater in the ventral subdivisions (dysgranular/agranular), there was area of overlap between the two inputs. *y*-axis: DV distance (μm); *x*-axis: normalized fluorescence intensity. ***E***, 60× magnification of a ROI in dysgranular GC from the slice shown in panel ***C***. Labeled fibers from VPMpc (green; bottom left), BLA (red; top right), counterstaining with fluorescent Nissl (blue; top left), and merge (bottom right).

The inputs from BLA and VPMpc to GC play a fundamental role in cortical taste processing. To begin to understand how VPMpc and BLA sculpt GC activity we used anterograde labeling of BLA and VPMpc projections and analyzed the distribution of their axonal fields in GC. AAV9 viral vectors expressing either GFP or RFP were bilaterally injected into VPMpc and BLA in a group of rats, with the fluorescent tag counterbalanced between subjects. Following two to three weeks of incubation, animals were transcardially perfused to harvest the brain; 100-μm slices were prepared, processed and imaged to determine the extent of the projections and quantify the overlap between inputs in GC. Representative slices showing injection sites for VPMpc (green soma) and BLA (red soma) and counterstained with Hoechst (blue nuclei) are shown in Figure 1*A*,*B*, respectively. Both BLA (Fig. 1*C*, red) and VPMpc (Fig. 1*C*, green) neurons sent direct axonal projections to GC. Slices for GC analysis were counterstained with fluorescent Nissl (blue soma) to better visualize layers and divisions (Fig. 1*C*,*E*). Afferents from VPMpc were mostly distributed in the granular and dysgranular subdivisions of GC, while axons from BLA were primarily distributed in the dysgranular and agranular subdivisions. To compare the DV distribution of both projections, we took advantage of the fluorescent tags marking the axonal fields. Using ImageJ, two linear ROIs were drawn orthogonally through the subdivisions of GC (Fig. 1*C*, Pir. lines). Each ROI extended 2000 μm from piriform cortex towards the somatosensory cortex (Som.), one across the superficial (ROI A) and one across the deep (ROI B) cortical layers. Fluorescence intensity was measured along each ROI from the border of Som. (0 μm on the *y*-axis; Fig. 1*D*) through granular, dysgranular and agranular GC, ending at the border of Pir. (2000 μm on the *y*-axis; Fig. 1*D*). Intensities were normalized to the maximum value measured for each input in each ROI in each slice, averaged in 50-μm bins, and the average ± SEM was plotted against distance for each bin (Fig. 1*D*). As shown in Figure 1*D*, VPMpc axons tend to be distributed more dorsally (toward Som.) and BLA axons appear more ventrally (toward Pir.), consistent with previous anatomic reports (Saper, 1982). VPMpc and BLA axonal fields showed the highest degree of overlap in the dysgranular subdivision of GC, although terminal fields from both inputs could be found in all subdivisions of GC (Fig. 1*E*). These data suggest that a group of neurons in GC may be GC neurons responding to both inputs.

### *In vivo* intracellular recordings from GC of urethane-anesthetized rats

**Figure 2.**
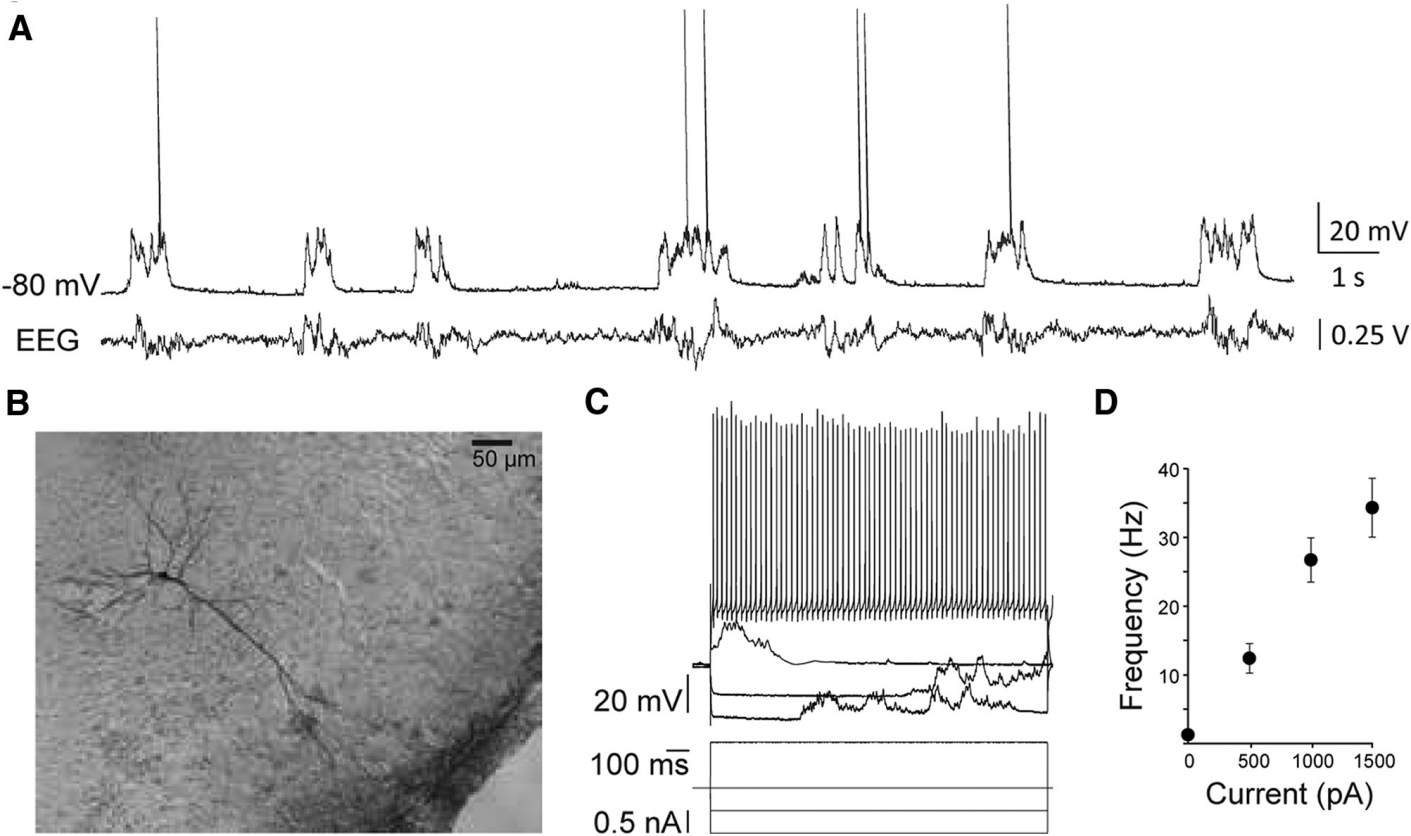
Intracellular recordings from pyramidal neurons in GC of urethane-anesthetized rats. ***A***, Representative trace of spontaneous activity recorded with a sharp electrode at resting membrane potential, −80 mV, and concurrent parietal EEG recording. ***B***, Reconstruction of a pyramidal cell filled with biocytin in dysgranular GC. ***C***, The cell from panel ***A***’s responses to injected hyperpolarizing and depolarizing DC current steps. ***D***, Mean ± SEM f/I curve for *n* = 26 cells. Firing properties observed for each cell resembled regular spiking pyramidal neurons. *y*-axis: firing frequency; *x*-axis: injected current (pA).

**Figure 3.**
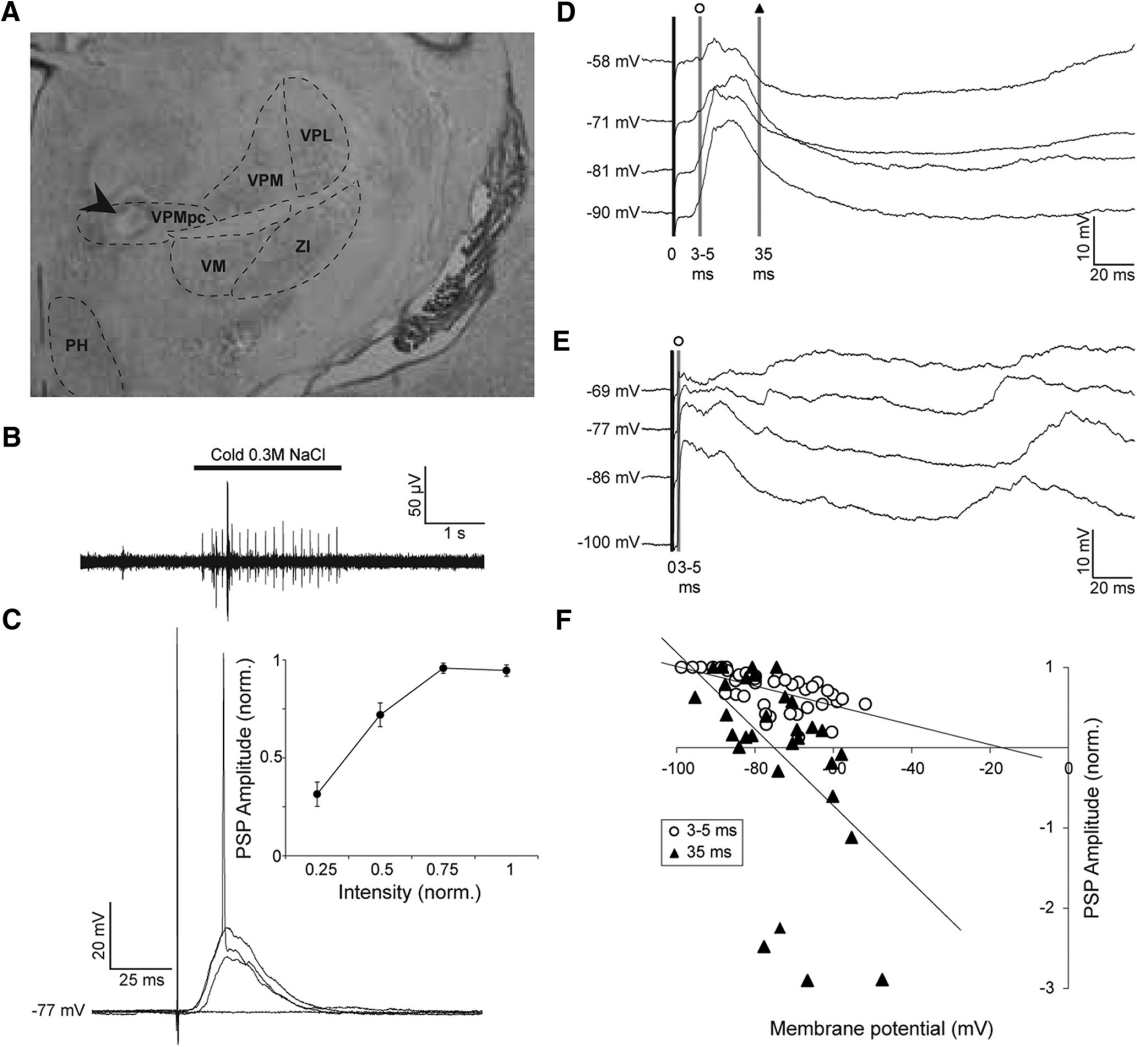
Stimulation of the gustatory thalamus evokes time-varying PSP in GC neurons. ***A***, Representative cresyl violet-stained coronal section showing the electrolytic lesion made by the tip of the stimulating electrode in VPMpc (arrow). VPMpc, ventroposteriomedial, parvocellular thalamus; VPM, ventroposteriomedial thalamus; VPL, ventroposteriolateral thalamus; VM, ventromedial thalamus; ZI, zona incerta; PH, posterior hypothalamus. ***B***, Representative trace of a multi-unit response to the application of cold 0.3 M NaCl to the oral cavity as recorded by the VPMpc stimulating electrode. For each animal, the final DV coordinate of the VPMpc electrode was determined by where the greatest response to tasting solution was found. In this example, the depth was −6.5 mm from the pial surface. ***C***, Representative synaptic response of a GC neuron to VPMpc stimulation at resting membrane potential, −77 mV. EPSP amplitude increases with intensity, resulting in an action potential at the greatest intensity. Inset, Mean ± SEM population intensity/response curve, normalized within each cell (*n* = 14). *y*-axis: normalized PSP amplitude; *x*-axis: normalized stimulation intensity. ***D***, Example trace of a cell responding to VPMpc stimulation with a monosynaptic EPSP followed by multi-synaptic inhibition, unveiled by stimulating with constant intensity at multiple holding potentials (*n* = 5/10). Shaded lines indicate the times post-PSP onset where amplitudes were measured for determining the reversal potential of the components of the PSP: open circle, 3–5 ms post-onset; black triangle, 35 ms post-onset. ***E***, Example trace of a cell responding to VPMpc stimulation with a monosynaptic EPSP followed by an upstate (*n* = 5/10). No inhibition could be unveiled by depolarizing holding potentials. Shaded line with open circle indicates amplitude was measured 3–5 ms post-PSP onset for determining reversal potential. ***F***, Population plot of reversal potentials for early component of PSP (open circles: 3–5 ms post-onset; *n* = 10) and late component (black triangles: 35 ms post-onset; *n* = 5). Reversal potentials were calculated by linear regression, represented by the lines on the graph. *y*-axis: normalized PSP amplitude; *x*-axis: holding potential (mV).

**Figure 4.**
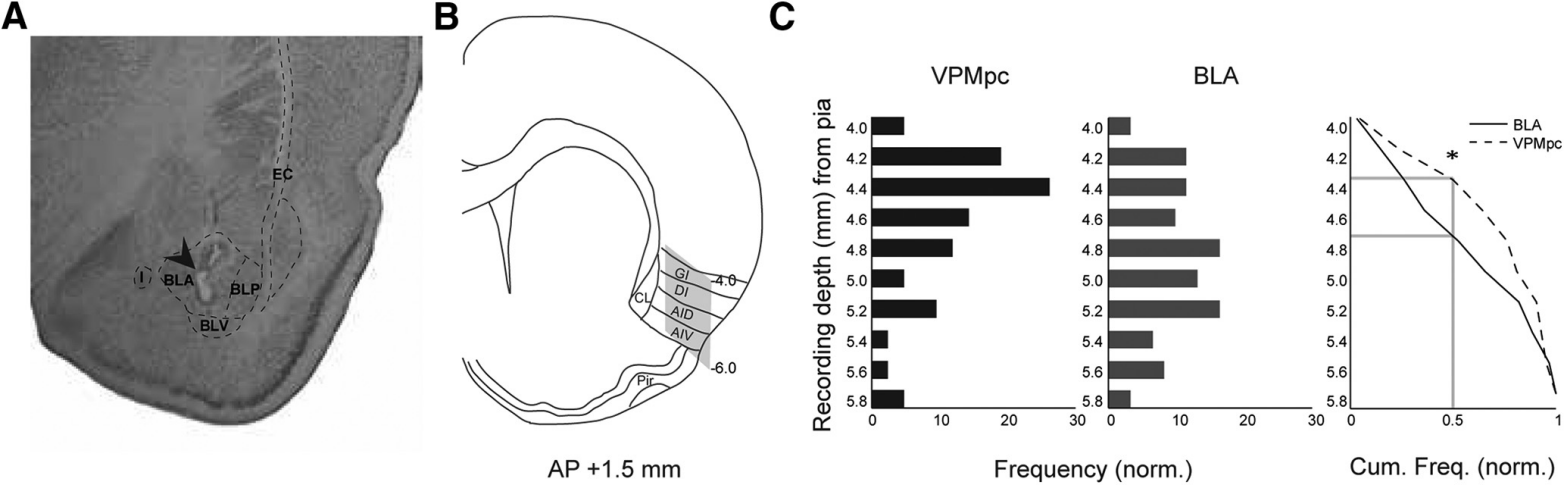
DV depth profile of GC neurons receiving input from VPMpc or BLA. ***A***, Representative cresyl violet-stained coronal section showing the electrolytic lesion made by the tip of the stimulating electrode in BLA (arrow). BLA, anterior BLA; BLP, posterior BLA; BLV, ventral BLA; I, intercalated nuclei of the amygdala; EC, external capsule. ***B***, Stereotaxic area in which we searched for GC cells for intracellular recording. The craniotomy was centered around AP + 1.5 mm, ML – 5.0 mm from bregma. Gray shading: search area, −4 to −6 mm ventral as measured from the pial surface, sampled with our recordings. Our search area covered all subdivisions of GC. CL., claustrum; GI, granular subdivision; DI, dysgranular subdivision; AID, dorsal agranular subdivision; AIV, ventral agranular subdivision. ***C***, Data were pooled as described in Materials and Methods, and the frequency distribution of cells responding to each stimulation type was plotted against the recording depth of the cell: VPMpc responsive (black bars; *n* = 42) and BLA responsive (gray bars; *n* = 61). Right panel, Cumulative sum of frequency comparing cells responding to BLA and VPMpc stimulation. *y*-axis: recording depth from pia, between −4 and −6 mm; *x*-axis: frequency. Asterisk indicates KS test *p* < 0.05.

Informed by the analysis of the dual anterograde tracing data, we asked whether BLA and VPMpc projections contact separate groups of GC neurons or whether there may be a population of neurons receiving directly activated by both projections. To relate the distribution of BLA and VPMpc axonal fields with functional synapses onto GC neurons, we employed *in vivo* intracellular recordings (Fig. 2) combined with electrical stimulation to resolve subthreshold synaptic events. First, we characterized the properties of VPMpc responses (Fig. 3). Then, we compared the DV position of recorded GC neurons that responded monosynaptically to VPMpc stimulation to our dataset of GC neurons that respond to BLA stimulation (Fig. 4). Neurons were recorded between 4.0 and 5.6 mm ventral from the pial surface, a range of depths indicating that this group of neurons was sampled across the entirety of GC.

### Identification of putative pyramidal neurons in GC

Once a cell was impaled and recordings stabilized, neurons were characterized with a series of procedures. First, spontaneous activity was recorded and assessed for 1 min to ensure stability; then square current steps of increasing amplitude were injected to determine the neuron firing pattern. Only regular spiking neurons, typically pyramidal neurons, were included in the analysis. Following these tests, stimulation of VPMpc or BLA at increasing stimulation intensities were delivered to test responsiveness and obtain input/output curves for either input. We initially focused on characterizing VPMpc response properties, as a detailed analysis of BLA input was reported in our previous study (Stone et al., 2011). Finally, after rapidly testing the input/output curve for BLA responses we begun assessing the effect of BLA stimulation on VPMpc evoked responses. Figure 2*A* shows the typical spontaneous activity of putative pyramidal neurons in GC recorded intracellularly under urethane anesthesia, paired with a simultaneously recorded EEG signal. The average downstate membrane potential and spontaneous firing rate observed at rest were −77 ± 1.5 mV and 0.98 ± 0.54 Hz, respectively. Histologic reconstruction of recorded neurons was performed at the end of each experiment. Figure 2*B* shows a biocytin-filled pyramidal neuron recorded at a depth of 5.35 mm from the pia and located in the intermediate layers of dysgranular GC. Neurons included in the analysis had an average input resistance of 33 ± 1 MΩ (Fig. 2*C*), consistent both with a previous study and with data from somatosensory cortical cells (Hasenstaub et al., 2007; Stone et al., 2011). For each neuron, the spike f/I relationship was assessed as further means to identify its putative pyramidal cell identity (Fig. 2*C*). The population f/I curve is shown in Figure 2*D*. In all 26 neurons included in the analysis, the firing rate increased with increasing steps of current, up to 33.6 ± 4.5 Hz at the maximum current tested, 1500 pA, and firing patterns resembled those of regular spiking neurons. Data were collected from two additional cells but discarded from analysis due to firing patterns and action potential widths resembling inhibitory neurons. Rarely, we recorded neurons with response properties resembling antidromic activation. These recordings were terminated and any data that was collected was not analyzed.

### Thalamus-evoked monosynaptic responses in GC neurons

To evoke thalamocortical responses in GC, a bipolar stimulating electrode was positioned in the VPMpc using stereotaxic coordinates and confirmed by mapping of taste responses in VPMpc (Fig. 3*A*,*B*; see Materials and Methods). For all preparations, the stimulating electrode was placed in the region that showed maximal single/multi-unit responsiveness to the delivery of cold 0.3 M NaCl solution to the oral cavity (Fig. 3*B*). The correct placement of the electrode was further confirmed with *post hoc* histology (Fig. 3*A*) indicating that electrodes positioned in the taste-responsive area of the thalamus were always within VPMpc (Fig. 3*A*). In our experimental configuration, we could reliably evoke taste responses within the range −6.0 to −6.8 mm DV from the pia (Fig. 3*B*), consistent with other reports (Verhagen et al., 2003; Liu and Fontanini, 2015). The average DV depth of the stimulating electrode in this study was 6.4 ± 0.04 mm.

GC neurons responded with a depolarizing PSP to electrical stimulation of VPMpc. PSP amplitude increased with increasing stimulation intensity (Fig. 3*C*). Most neurons, 71% (*n* = 10/14), could be driven to fire an action potential at a maximal intensity. The average stimulation intensity to evoke the largest subthreshold PSP was 0.65 ± 0.15 mA, with a range of 0.1–2.25 mA. At resting membrane potential, subthreshold peak PSP amplitudes averaged 11.7 ± 2.0 mV, with an average onset latency of 4.1 ± 0.8 ms and average time-to-peak of 19.7 ± 3.7 ms. Delays from stimulus onset are consistent with monosynaptic responses. We then examined the voltage dependency of VPMpc-PSPs. To do that, we used the stimulation intensity evoking the largest subthreshold PSP and delivered stimuli while injecting a steady state current into the cell to progressively vary the membrane potential from hyperpolarized to depolarized (Fig. 3*D*,*E*). In all neurons tested (*n* = 10), the amplitude of the early component of the response was measured 3–5 ms post-PSP onset. At a depolarized holding potential, where the driving force of the cell is maximal for inhibition and reduced for excitation, the size of this early component was 75 ± 10% of the baseline response measured at resting potential (baseline V_m_: −77 ± 2 mV; PSP amplitude, baseline V_m_: 4.4 ± 1.8 mV; depolarized V_m_: −63 ± 2 mV; PSP amplitude, depolarized V_m_: 2.8 ± 1.2 mV; PSP amplitude depolarized vs baseline, paired samples one-tailed *t* test: *p* = 0.03). At hyperpolarized holding potentials, where the driving force is maximal for excitation, the early component was 164 ± 18% of the baseline PSP measured at rest (hyperpolarized V_m_: −94 ± 2 mV; PSP amplitude, hyperpolarized V_m_: 6.8 ± 2.6 mV; PSP amplitude hyperpolarized vs baseline, paired samples one-tailed *t* test: *p* = 0.04).

We assessed the reversal potential of the early and late component of the VPMpc-evoked responses by normalizing PSP amplitudes to the largest evoked response within each neuron and plotting these values against their respective holding potential. The PSP reversal potential was quantified as the *x-intercept* of the linear regression of the amplitude/holding potential plot (Fig. 3*F*). The early PSP component reversed at −16.8 mV. Given the constraints of intracellular recording techniques this reversal potential is close to that of AMPA receptors, consistent with the presence of a monosynaptic glutamatergic projection from VPMpc to GC (Moore and Nelson, 1998; Stone et al., 2011). While the initial component of the evoked PSP was uniformly excitatory in all recorded neurons, two distinct groups could be identified based on the later part of the VPMpc-evoked response. One group of neurons, 50% (*n* = 5/10), showed a PSP with a late component reversing at −75.2 mV (Fig. 3*D*), near the reversal potential for chloride, indicative of an early glutamatergic component followed by a GABA_A_-mediated polysynaptic response (Maffei et al., 2006; Stone et al., 2011). The remaining 50% of GC neurons (Fig. 3*E*) did not show an inhibitory component in the late phase of the VPMpc-PSP.

We further compared the early component of the PSP between GC neurons with and without polysynaptic inhibition and found that in neurons lacking the inhibitory component (Fig. 3*E*), the initial depolarizing component of the response reversed at −6.5 mV, more depolarized than the population average, suggesting that this population of neurons showed a predominantly excitatory response to VPMpc stimulation. In contrast, the early component of the PSP for neurons with a late inhibitory component reversed at −29.0 mV, reflecting a mixed response likely due to early recruitment of inhibition.

Neurons receiving polysynaptic inhibition did not differ from neurons lacking a late inhibitory component in terms of: DV position (polysynaptic inhibition group: 4.6 ± 0.3 mm; no inhibition group: 4.3 ± 0.1 mm; independent samples two-tailed *t* test, *p* = 0.29), peak amplitude at V_m_ (polysynaptic inhibition group: 6.4 ± 0.8 mV; no inhibition group: 7.0 ± 2.9 mV; independent samples two-tailed *t* test, *p* = 0.84), onset latency (polysynaptic inhibition group: 8.0 ± 2.7 ms; no inhibition group: 2.5 ± 0.4 ms; independent samples two-tailed *t* test, *p* = 0.08), or time-to-peak (polysynaptic inhibition group: 34.6 ± 13.7 ms and no inhibition group: 18.1 ± 6.9 ms, respectively; independent samples two-tailed *t* test, *p* = 0.31). These data suggest that stimulation of the VPMpc can evoke a PSP with distinct ratios of excitation and inhibition, possibly reflecting distinct recruitment of excitatory and inhibitory circuits by thalamocortical afferents.

### DV profile of GC neurons responding to VPMpc or BLA stimulation

In our experiments, we monitor the depth of the sharp electrode tip and record from GC cells located 4–6 mm ventral from the pial surface. Thus, our recordings sample broadly within the taste-responsive region of the insular cortex (Fig. 4*B*, shaded region of stereotaxic reconstruction). We had previously characterized the response properties of GC neurons responding to BLA stimulation (Stone et al., 2011). As now we have also identified GC neurons responding to VPMpc stimulation, we used information about the positions of the recordings from the previous study and the current work to map the locations of GC cells responding to either input, and compare that to the anterograde tracing data. The depth profile of recordings of monosynaptic responses to VPMpc or BLA stimulation is shown in Figure 4*C*. For neurons responding to BLA, the plot includes the positioning of neurons recorded in this study pooled with neurons included in our previous work (Stone et al., 2011) and with neurons that were tested for responsiveness, but with recording duration insufficient to contribute to the other analyses (*n* = 50; see Materials and Methods). Figures 3*A*, 4*A* show histologic verifications of stimulating electrodes in VPMpc and BLA.

Neurons responding to VPMpc or BLA stimulation could be found at all depths between −4 and −5.8 mm from the pia, which is the full extent of our search region in GC as guided by the stereotaxic atlas (Fig. 4*B*). However, the positioning of GC neurons responding with monosynaptic PSPs to VPMpc stimulation showed a dorsal bias (Fig. 4*C*, left panel; average DV depth 4.6 ± 0.07 mm; *n* = 42); while neurons responding with monosynaptic PSPs to BLA stimulation tended to be more ventral (Fig. 4*C*, middle panel; average DV depth 4.8 ± 0.06 mm; *n* = 61). The cumulative sum of frequency plot in Figure 4*C*, right panel, shows that the differences in depth profiles were significant (KS test applied to the BLA responding and VPMpc responding groups, D = 0.2865, *p* = 0.03). Thus, the depth profile of our electrophysiology data are congruent with our anatomic data (Fig. 1) and with data from previous studies suggesting that VPMpc projects primarily to the more dorsal granular and dysgranular subdivisions (Saper, 1982; Kosar et al., 1986; Allen et al., 1991; Nakashima et al., 2000; Verhagen et al., 2003), while BLA projects primarily to the more ventral dysgranular and agranular subdivisions (Saper, 1982; Allen et al., 1991; Hanamori, 2009). However, the presence of neurons responding to both inputs across the full extent of GC suggests that inputs are not segregated and that a significant degree of functional overlap is expected.

### Monosynaptic inputs from VPMpc and BLA converge on a subpopulation of GC neurons

**Figure 5.**
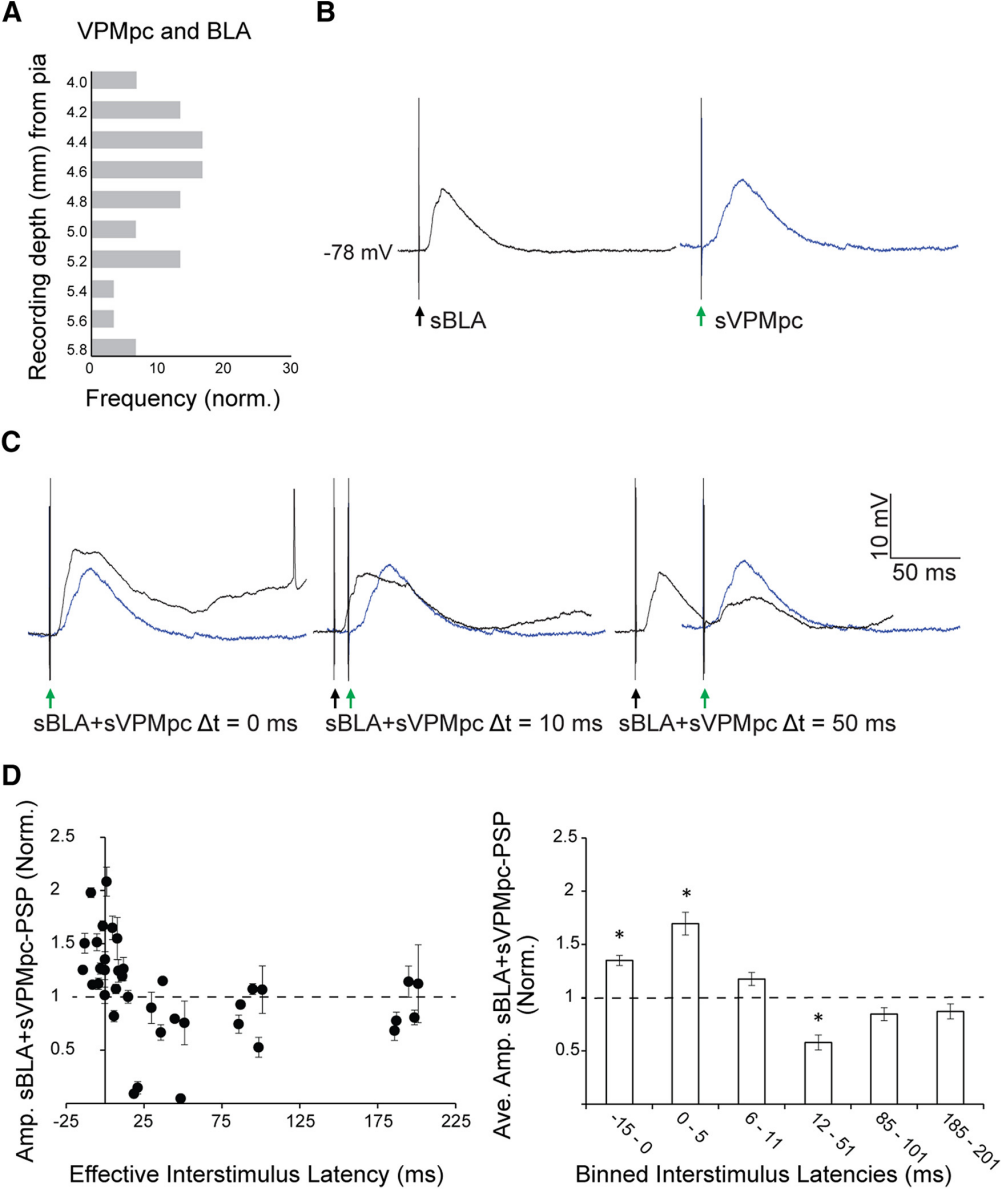
Time-dependent modulation of VPMpc-PSPs by preceding BLA stimulation. ***A***, Frequency distribution of cells responsive to both BLA and VPMpc plotted against recording depth (light gray bars; *n* = 30). *y*-axis: recording depth from pia, between −4 and −6 mm; *x*-axis: frequency. ***B***, Example traces recorded at resting membrane potential, −78 mV, from a GC cell 4.4 mm ventral from the pial surface. Left, black trace, Evoked PSP to BLA stimulation alone (sBLA). Black arrow indicates BLA stimulation onset. Right, blue trace, Evoked PSP to VPMpc stimulation alone (sVPMpc). Green arrow indicates VPMpc stimulation onset. ***C***, Evoked PSPs in the same cell for a single BLA shock followed by a single VPMpc shock (sBLA+sVPMpc) at latencies of 0, 10, and 50 ms (black traces). Superimposed blue traces reflect the evoked PSP at baseline (sVPMpc), as in panel ***A***, to facilitate comparing PSP amplitudes. This cell exhibits the typical response pattern: enhanced PSP amplitude at short latency and shunted PSP amplitude at longer latency. ***D***, Population data from *n* = 5 cells tested. Left panel, Normalized amplitudes of evoked sBLA+sVPMpc-PSP at varying interstimulus latencies. *y*-axis: normalized mean ± SEM PSP amplitude; *x*-axis: effective interstimulus latency (ms). Right panel, Effective interstimulus latency was binned and sBLA+sVPMpc-PSP amplitudes from the population of cells were averaged. *y*-axis: normalized mean ± SEM PSP amplitude; *x*-axis: binned interstimulus latency (ms). Asterisks indicate *p* < 0.01.

Informed by the anatomic analysis and depth profile of VPMpc and BLA responsive neurons, we asked whether there is evidence of convergent inputs from these regions onto a population of GC neurons. To address this question, we implanted stimulating electrodes in both VPMpc and BLA in a subset of animals and performed intracellular recordings to assess neuronal responsiveness to stimulation of either (or both) inputs. We identified a group of neurons that responded to both inputs with delays consistent with monosynaptic connections from both regions (*n* = 30). GC neurons responding monosynaptically to both VPMpc and BLA stimulation were distributed throughout the depth profile sampled (Fig. 5*A*; average DV depth: 4.7 ± 0.09 mm; *n* = 30). These data indicate that VPMpc and BLA afferents not only overlap within the same region of GC but can also target the same postsynaptic neurons. In addition, the data suggest that the functional overlap of the inputs is more extensive than expected from tracing studies alone and is not limited to a specific subdivision of GC.

Next, we investigated the dynamics of integration of VPMpc and BLA in a group of GC neurons receiving convergent inputs. Experimental evidence shows that VPMpc inactivation almost completely abolishes taste coding in GC, while BLA inactivation affects taste processing without dramatically impairing it (Piette et al., 2012; Samuelsen et al., 2013). According to the definition of “driver” and “modulator” input (Sherman and Guillery, 1998), we interpreted published results as evidence that the VPMpc drives GC, while BLA modulates it. Therefore, we investigate the effect of preceding BLA stimulation on VPMpc responses. We previously reported that BLA-PSPs have time varying dynamics (Stone et al., 2011). Thus, we hypothesized that BLA could modulate pyramidal neurons’ responsiveness to thalamic input differently depending on the timing of BLA and VPMpc stimulation. As the initial portion of BLA and VPMpc responses are both dominated by excitation, we predicted that if the latency between BLA and VPMpc stimuli was sufficiently short the early excitatory components of both PSPs could sum, enhancing neurons’ response amplitudes. Conversely, we expected a suppression of VPMpc-PSPs if VPMpc was stimulated during the inhibitory phase of the BLA-PSP. To test this possibility, we adjusted the stimulation intensity to evoke the largest subthreshold PSPs for both inputs (Fig. 5*B*), then delivered a single baseline VPMpc stimulus (sVPMpc) followed, 10 s later, by a pairing in which a single BLA stimulus (sBLA) preceded a VPMpc stimulus (sBLA+sVPMpc). The amplitude of the PSP resulting from VPMpc and BLA pairing (sBLA+sVPMpc-PSP) was compared with that of the baseline PSP (sVPMpc-PSP). The interstimulus latency between BLA and VPMpc stimulation in the pairing was varied between 0 and 200 ms (Fig. 5*C*). For the neurons in this group (*n* = 5), peak sBLA-PSP amplitude was 9.0 ± 2.5 mV, with an onset latency of 10.2 ± 2.3 ms, and peak sVPMpc-PSP amplitude was 8.6 ± 1.3 mV, with an onset latency of 3.7 ± 1.2 ms.

While all VPMpc and BLA evoked responses showed onset delays compatible with monosynaptic connectivity, the time of PSP onset for the two PSPs could show some degree of variability in different neurons. Thus, for each cell the effective interstimulus latency of the sBLA+sVPMpc pairing was corrected by the difference between the onset latencies of the PSPs evoked by sBLA and sVPMpc stimulation alone. Once corrected, the effective range of timing intervals tested was between −15 and 201 ms.

Pairing of sBLA and sVMPpc stimuli modulated the amplitude of the resulting sBLA+sVPMpc-PSP in a time-dependent fashion as predicted (Fig. 5*C*,*D*). In all cells tested with this protocol (*n* = 5), when sBLA was paired with sVPMpc (sBLA+sVPMpc) at effective latencies between −15 and 5 ms, the amplitude of the evoked PSP was significantly larger than that of the baseline sVPMpc-PSP (Fig. 5*D*; normalized PSP amplitude sBLA+sVPMpc, Δt −15 to 0 ms: 140 ± 5%; Δt 0–5 ms: 170 ± 10%; paired sample two-tailed *t* tests, *p* = 2 × 10^−6^ and *p* = 0.001, respectively). If sBLA preceded sVPMpc by 6–11 ms, there was no significant modulation of the VPMpc response (Fig. 5*D*; normalized PSP amplitude Δt 6–11 ms: 120 ± 6%; paired sample two-tailed *t* test, *p* = 0.05). As the time window for the pairing of BLA and VPMpc stimulation widened to 12–51 ms, the amplitude of the PSP evoked by the pairing was significantly decreased compared with baseline (Fig. 5*D*; normalized PSP amplitude, Δt 12–51 ms: 60 ± 7%; paired sample two-tailed *t* test, *p* = 3 × 10^−4^). Finally, when sVPMpc followed sBLA by 85–201 ms, there was no significant modulation of the resulting PSP (Fig. 5*D*; normalized PSP amplitude, Δt 85–101 ms: 85 ± 6%; Δt 185–201 ms: 87 ± 7%, paired sample two-tailed *t* tests, *p* = 0.15 and *p* = 0.05, respectively).

These data suggest that excitation from BLA and VPMpc inputs can sum maximally when inputs arrive with delays shorter than 5 ms. Because of the early emergence of the inhibitory component of the sBLA-PSP, excitation no longer sums by latencies of 6–11 ms, and inhibitory shunting of the evoked sBLA+sVPMpc-PSP occurs at latencies of 12–51 ms. At latencies longer than 85 ms, a single BLA stimulus is not effective at modulating sVPMpc-PSPs. Thus, BLA inputs to GC can significantly modulate incoming thalamocortical responses, supporting the important role for BLA in the processing of taste information.

### Twenty-Hertz BLA bursts modulate GC responses to VPMpc stimulation

**Figure 6.**
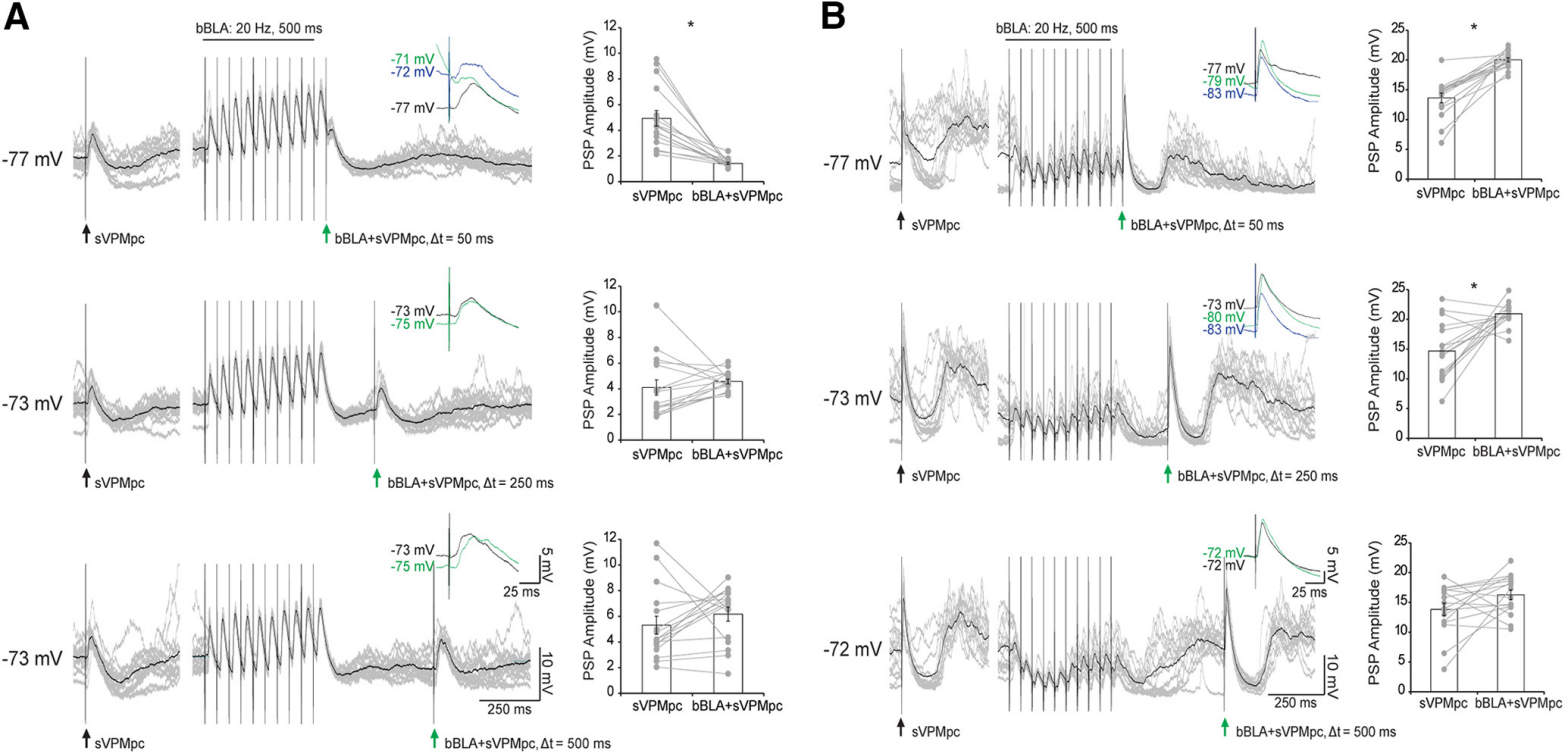
Two populations of GC neurons with distinct BLA modulation of VPMpc-PSPs. ***A***, top left panel, Traces from a cell representing the group of *n* = 5/8 that show suppressed VPMpc-PSP amplitude following a BLA burst, recorded −4.2 mm ventral to pia. A single VPMpc stimulus (sVPMpc; black arrow) was delivered under baseline conditions; 500–1500 ms later, a 500-ms, 20-Hz BLA burst (bBLA) was delivered and then followed by a VPMpc stimulus (bBLA+sVPMpc; green arrow) at a latency of 50 ms (gray traces: 16 overlaid trials; black trace: average of 16 trials). Inset, Close-up comparison of VPMpc-PSP amplitudes evoked under baseline conditions (black trace), 50 ms after a BLA burst (green trace), and at a comparable V_m_ to the post-burst trace, maintained by DC current injection (blue trace). Top right panel, Raw data for peak VPMpc-PSP amplitudes (gray circles) and mean ± SEM (white rectangles) for the traces pictured. Lines connect amplitudes recorded during the same trial. Asterisks indicate *p* < 0.01. *y*-axis: PSP amplitude (mV); *x*-axis: sVPMpc and bBLA+sVPMpc. Middle panel, bBLA+sVPMpc latency of 250 ms. Bottom panel, bBLA+sVPMpc latency of 500 ms. ***B***, top left panel, Traces from a cell representing the group of *n* = 3/8 that show increased VPMpc-PSP amplitude following a BLA burst, recorded −4.6 mm ventral to pia. A single VPMpc stimulus (sVPMpc; black arrow) was delivered under baseline conditions; 500–1500 ms later, a 500-ms, 20-Hz BLA burst (bBLA) was delivered and then followed by a VPMpc stimulus (bBLA+sVPMpc; green arrow) at a latency of 50 ms (gray traces: 16 overlaid trials; black trace: average of 16 trials). Inset, Close-up comparison of VPMpc-PSP amplitudes evoked under baseline conditions (black trace), 50 ms after a BLA burst (green trace), and at a comparable V_m_ to the post-burst trace, maintained by DC current injection (blue trace). Top right panel, Raw data for peak VPMpc-PSP amplitudes (gray circles) and mean ± SEM (white rectangles) for the traces pictured. Lines connect amplitudes recorded during the same trial. Asterisks indicate *p* < 0.01. *y*-axis: PSP amplitude (mV); *x*-axis: sVPMpc and bBLA+sVPMpc. Middle panel, bBLA+sVPMpc latency of 250 ms. Bottom panel, bBLA+sVPMpc latency of 500 ms.

**Figure 7.**
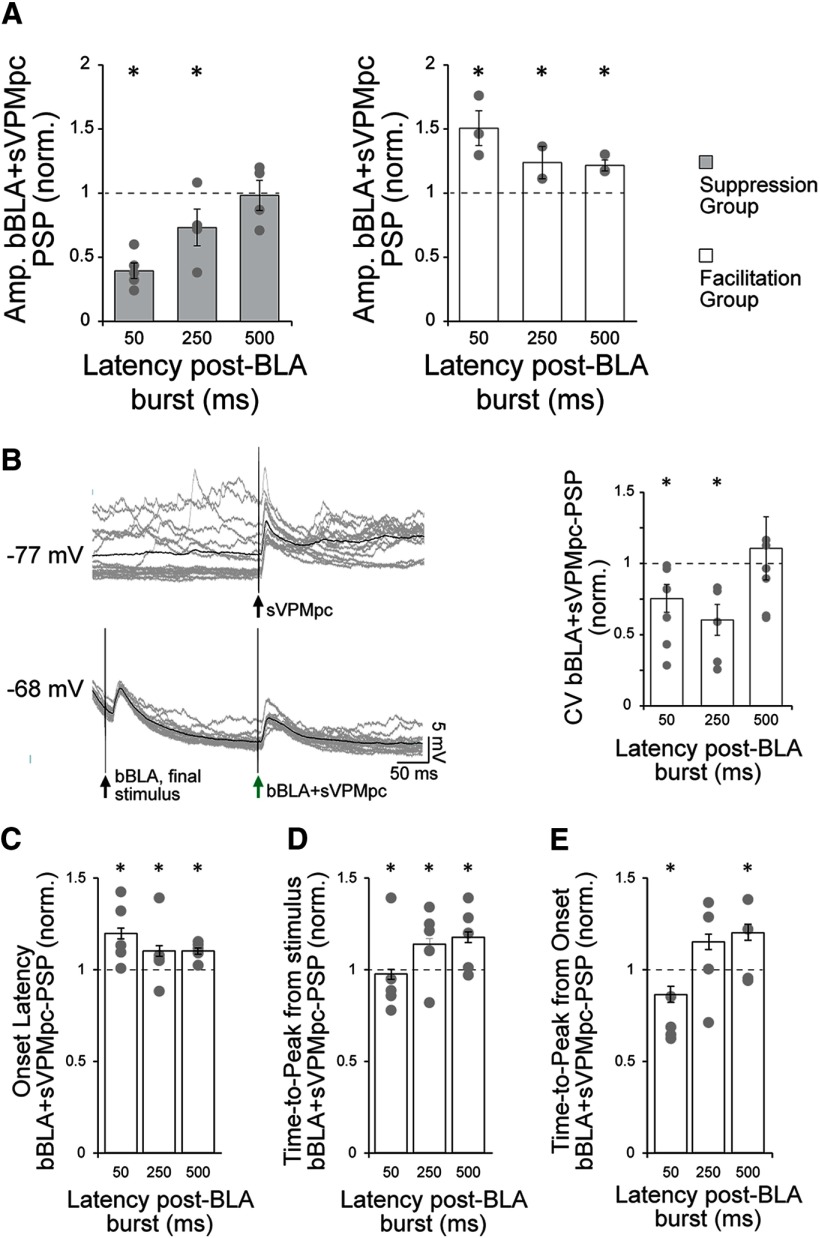
BLA bursts modulate VPMpc-PSP amplitude and temporal dynamics. ***A***, For each cell and post-BLA burst latency tested, the average amplitude of the evoked VPMpc-PSP under baseline conditions (sVPMpc) was used to normalize the amplitude of the evoked post-burst PSP (bBLA+VPMpc). These normalized amplitudes were then averaged across cells with the same response pattern and plotted. Left panel, Group of *n* = 5/8 cells (as in Fig. 6*A*), where the VPMpc-PSP was depressed following a BLA burst. Each cell’s mean, normalized PSP amplitude (gray circles) and the group mean ± SEM (light gray bars) is shown for each latency tested. Asterisks indicate *p* < 0.01. Right panel, Group of *n* = 3/8 cells (as in Fig. 6*B*), where the VPMpc-PSP was enhanced following a BLA burst. Each cell’s mean, normalized PSP amplitude (gray circles) and the group mean ± SEM (white bars) is shown for each latency tested. Asterisks indicate *p* < 0.01. *y*-axis: normalized bBLA+sVPMpc-PSP amplitude; *x*-axis: post-BLA burst latency (ms). ***B***, left panel, Representative trace from a GC cell recorded 4.8 mm ventral from the pia to VPMpc stimulation under control conditions (sVPMpc; top trace) and 250 ms following a BLA burst (bBLA+sVPMpc, bottom trace). Gray traces: 16 overlaid trials. Black trace: Average of 16 trials. Right panel, CV was calculated from the peak PSP amplitudes evoked by VPMpc stimulation at post-BLA burst latencies of 50, 250, and 500 ms and normalized to the CV calculated under baseline conditions. Each cell’s mean, normalized CV (*n* = 8; gray circles) and group mean ±SEM (white bars) are shown. Asterisks indicate *p* < 0.05. *y*-axis: normalized CV for bBLA+sVPMpc-PSP; *x*-axis: post-BLA burst latency (ms). ***C***, VPMpc-PSP onset latency was measured under baseline conditions (sVPMpc) and following a BLA burst (bBLA+sVPMpc) at latencies of 50, 250, and 500 ms. The PSP onset latency was significantly increased at each post-burst interval as compared with baseline, with the strongest effect at 50 ms. Asterisks indicate *p* < 0.05. *y*-axis: normalized bBLA+sVPMpc-PSP onset latency; *x*-axis: post-BLA burst latency (ms). ***D***, VPMpc-PSP time-to-peak was measured from stimulus onset under baseline conditions (sVPMpc) and following a BLA burst (bBLA+sVPMpc) at latencies of 50, 250, and 500 ms. The PSP time-to-peak was significantly smaller at a post-burst latency of 50 ms and larger at 250- and 500-ms latencies. Asterisks indicate *p* < 0.05. *y*-axis: normalized bBLA+sVPMpc-PSP time-to-peak from stimulus onset; *x*-axis: post-BLA burst latency (ms). ***E***, VPMpc-PSP time-to-peak was measured from PSP onset under baseline conditions (sVPMpc) and following a BLA burst (bBLA+sVPMpc) at latencies of 50, 250, and 500 ms. The PSP time-to-peak was significantly smaller at a post-burst latency of 50 ms and larger at 500-ms latency. Asterisks indicate *p* < 0.05. *y*-axis: normalized bBLA+sVPMpc-PSP time-to-peak from PSP onset; *x*-axis: post-BLA burst latency (ms).

A recent study showed that when rats learn to press a lever to self-administer tastes following an auditory cue, cue-responsive BLA neurons fire at 20 Hz (Samuelsen et al., 2012). We have previously shown that when 20-Hz trains of BLA electrical stimuli are delivered, both excitation and inhibition are recruited and summate in GC cells over the course of the stimulus. Whether excitation or inhibition dominates depends largely on the membrane potential of the recorded neuron (Stone et al., 2011). Here, we examined the effect of preceding BLA bursts at a behaviorally relevant frequency on VPMpc-PSPs onto GC neurons. A train of BLA stimuli (bBLA) at 20 Hz (500-ms duration) was delivered 50, 250, and 500 ms prior to a single VPMpc stimulus (sVPMpc). The amplitude of the PSP evoked by the bBLA and sVPMpc pairing (bBLA+sVPMpc) was compared with that of a baseline sVPMpc-PSP (Fig. 6). This stimulation protocol is modified from a study of prefrontal suppression of excitatory afferents to the ventral striatum (Calhoon and O'Donnell, 2013). As shown in the examples in Figure 6*A*,*B*, and the population data summarized in Figure 7*A*, we tested *n* = 8 GC cells in this protocol and identified two groups of neurons whose VPMpc-evoked responses are differentially modulated by a preceding BLA burst.

A group of neurons (Figs. 6*A*, 7*A*, left panel; *n* = 5/8 or 63% of neurons tested) showed significant suppression of the bBLA+sVPMpc-PSP amplitude in a time dependent manner while the second group of neurons showed facilitation (Figs. 6*B*, 7*A*, right panel; *n* = 3/8 or 38%). In both cases, the membrane potential of the recorded neuron was modulated by the BLA burst, so we first assessed whether the effects of the pairing could be explained by the state of depolarization/hyperpolarization of the neuron.

In the suppression group (Fig. 6*A*), there was a significant shift toward a more depolarized membrane potential at the time of the post-burst VPMpc stimulus (V_m_ 50 ms post-burst latency, sVPMpc: −78.4 ± 0.5 mV; bBLA+sVPMpc: −76.3 ± 0.4 mV; paired sample two-tailed *t* test: *p* = 2 × 10^−4^). To control for the possibility that the suppression of sVPMpc-PSP amplitude resulted from altered ionic conductance due to the depolarization, we compared the PSP evoked by bBLA+sVPMpc pairing to the sVPMpc-PSPs evoked in a voltage-matched condition where DC current was injected to maintain a comparable depolarized holding potential (Fig. 6*A*, inset, green trace vs blue trace). In the voltage-matched conditions, the average V_m_ at the time of VPMpc stimulus delivery, −75.0 ± 0.6 mV, did not differ significantly from that of the bBLA+sVPMpc pairing, −76.3 ± 0.4 mV (independent samples two-tailed *t* test: *p* = 0.07). For all cells, the evoked voltage-matched-sVPMpc-PSP amplitude was larger than that of the corresponding bBLA+sVPMpc-PSP (PSP amplitude voltage-matched-sVPMpc: 6.7 ± 1.0 mV; bBLA+sVPMpc: 3.1 ± 0.3 mV; independent samples two-tailed *t* test: *p* = 10^−4^). This finding indicates that the suppressing effect of preceding BLA bursts on VPM-PSPs was independent of a shunting of excitatory drive induced by membrane depolarization.

An additional set of controls was performed to test whether the facilitation observed in the second group of neurons (Fig. 6*B*) could be explained by a modulation of V_m_ by the BLA burst. For this group of neurons, we first compared the amplitude of bBLA+sVPMpc-PSP and baseline sVPMpc-PSP during trials where the baseline VPMpc stimulation was delivered during the downstate of the ongoing membrane potential oscillation (downstate-sVPMpc). The amplitude of the evoked sVPMpc-PSP was larger during downstate trials, as compared with upstate trials, due to increased excitatory drive. When analysis of bBLA+sVPMpc-PSP amplitude was restricted to downstate-sVPMpc trials, the downstate-sVPMpc-PSP was still significantly smaller than that of bBLA+sVPMpc-PSPs at all pairing delays tested (50-ms PSP amplitude, downstate-sVPMpc: 7.6 ± 0.8 mV; bBLA+sVPMpc: 10.7 ± 0.9 mV; 250-ms PSP amplitude, downstate-sVPMpc: 8.3 ± 1.5 mV; bBLA+sVPMpc: 9.5 ± 1.7 mV; 500-ms PSP amplitude, downstate-sVPMpc: 4.8 ± 0.7 mV; bBLA+sVPMpc: 6.0 ± 0.9 mV; paired sample two-tailed *t* tests, *p* = 2 × 10^−12^, *p* = 0.03, and *p* = 0.002, respectively). In order to further control for any effect of a shift in membrane potential on the facilitation of the VPMpc-PSP induced by a preceding BLA burst, a second comparison was drawn between the amplitude of the bBLA+sVPMpc-PSPs at a 50-ms latency to that of PSPs evoked by VPMpc stimulation delivered in a voltage-matched condition. For each cell, the average V_m_ at the time of bBLA+sVPMpc stimulus delivery was measured and evoked PSP amplitudes were compared with trials in which current was injected to maintain a comparable V_m_ during sVPMpc stimulation (voltage-matched-sVPMpc). As shown in the inset of Figure 6*B*, the PSP evoked during a voltage-matched trial (blue trace) is smaller in amplitude than the bBLA+sVPMpc-PSP (green trace), despite the more hyperpolarized membrane potential at stimulus onset in this case. This result was also reflected in the group data, although when pooled, the pre-stimulus V_m_ of the sVPMpc-PSP and bBLA+sVPMpc-PSP did not differ significantly (Pre-VPMpc stimulus V_m_, sVPMpc: −70.3 ± 0.9 mV; bBLA+sVPMpc: −69.9 ± 0.8 mV; paired sample two-tailed *t* test, *p* = 0.5). In the voltage-matched condition (Pre-VPMpc stimulus V_m_, voltage-match-sVPMpc: −68.8 ± 1.1 mV; bBLA+sVPMpc: −69.9 ± 0.8 mV; independent sample two-tailed *t* test, *p* = 0.33) the amplitude of the evoked sVPMpc-PSP was significantly smaller than that evoked by bBLA+sVPMpc pairing at 50-ms latency (PSP amplitude, voltage-matched-sVPMpc: 8.3 ± 1.1 mV; bBLA+ sVPMpc: 11.6 ±; 0.9 mV; independent sample two-tailed *t* test: *p* = 0.03). Thus, the modulation of the VPMpc-PSP by BLA is not due to a shift in V_m_ but depends on the integration of synaptic inputs.

For both groups of neurons, we analyzed the time dependency of the suppression and facilitation. The strongest suppression was observed at the shortest latency (50 ms), with the bBLA+sVPMpc-PSP amplitude accounting for 40 ± 2% of the baseline sVPMpc-PSP on average (Figs. 6*A*, 7*A*, left panel; PSP amplitude, sVPMpc: 8.5 ± 0.7 mV; bBLA+sVPMpc: 3.4 ± 0.3 mV; *n* = 5; paired sample two-tailed *t* test, *p* = 10^−14^). At a latency of 250 ms, bBLA+sVPMpc-PSPs were 77 ± 4% of baseline sVPMpc-PSPs (Figs. 6*A*, 7*A*, left panel; PSP amplitude, baseline sVPMpc: 9.0 ± 0.8 mV; bBLA+sVPMpc: 6.6 ± 0.5 mV; *n* = 5; paired sample two-tailed *t* test, *p* = 3 × 10^−5^). At a latency of 500 ms, the modulatory effect of the BLA burst was no longer significant (Figs. 6*A*, 7*A*, left panel; PSP amplitude, baseline sVPMpc: 7.9 ± 0.6 mV; bBLA+sVPMpc: 7.4 ± 0.5 mV; *n* = 5; paired sample two-tailed *t* test, *p* = 0.3). These data suggest that the shunting effect of trains of BLA stimuli at 20 Hz on VPMpc responses is strongest for convergent inputs being activated within a short time window.

The enhancement of the sVPMpc-PSP in the facilitation group was observed at all bBLA+sVPMpc intervals tested (Figs. 6*B*, 7*A*, right panel), although the strongest effect was at a latency of 50 ms. In this group, the amplitude of the bBLA+sVPMpc-PSP averaged 151 ± 4% of the amplitude of the baseline sVPMpc-PSP when the BLA burst preceded the VPMpc stimulus by 50 ms (Figs. 6*B*, 7*A*, right panel; PSP amplitude, sVPMpc: 8.0 ± 0.7 mV; bBLA+sVPMpc: 11.6 ± 0.9 mV; *n* = 3; paired sample two-tailed *t* test, *p* = 10^−10^). The enhancement of the PSP relative to baseline remained significant for bBLA+ sVPMpc stimuli paired at 250–500 ms. For 250-ms intervals, the amplitude of the bBLA+sVPMpc-PSP averaged 123 ± 3% of the baseline sVPMpc-PSP (Figs. 6*B*, 7*A*, right panel; PSP amplitude, sVPMpc: 9.4 ± 1.2 mV; bBLA+sVPMpc: 12.3 ± 1.5 mV; *n* = 3; paired sample two-tailed *t* test, *p* = 0.001) and at 500 ms, the bBLA+ sVPMpc-PSP amplitude remained significantly above (122 ± 3%) that of baseline sVPMpc-PSPs (Figs. 6*B*, 7*A*, right panel; PSP amplitude, sVPMpc: 6.9 ± 0.8 mV; bBLA+sVPMpc: 8.2 ± 0.9 mV; *n* = 3; paired sample two-tailed *t* test, *p* = 0.006).

### Synaptic interactions underlie the modulatory effect of BLA on VPMpc-PSPs

We further analyzed the properties of the recorded GC neurons and the temporal dynamics of the synaptic responses to identify factors that could differentiate the modulatory effect of BLA bursts on VPMpc responses. Neurons showing a positive or negative modulation of sVPMpc-PSP amplitude by trains of BLA stimulation did not differ in depth of recording from the pia (enhancement group: 4.5 ± 0.2 mm; suppression group: 4.5 ± 0.1 mm; independent sample two-tailed *t* test, *p* = 0.90), maximum BLA-PSP amplitude during the 20-Hz BLA burst (enhancement group: 7.0 ± 1.3 mV; suppression group: 6.0 ± 1.7 mV; independent sample two-tailed *t* test, *p* = 0.60), peak amplitude of the excitatory component of the PSP evoked by a single baseline VPMpc stimulus recorded at resting membrane potential (enhancement group: 8.6 ± 3.4 mV; suppression group: 9.9 ± 2.2 mV; independent sample two-tailed *t* test, *p* = 0.77), or peak amplitude of the excitatory component of the PSP evoked by a single BLA stimulus at resting membrane potential (enhancement group: 5.0 ± 1.8 mV; suppression group: 5.3 ± 1.6 mV; independent sample two-tailed *t* test, *p* = 0.92).

Several additional response properties of sVPMpc-PSPs were modulated by preceding BLA bursts, although these effects were similar between the enhanced and suppressed groups. The membrane potential of GC neurons showed slow wave oscillations under urethane anesthesia (Fig. 1*A*) and VPMpc stimulation, delivered randomly during the neuron’s oscillations, can occur when the membrane is either in an up or down state. As shown previously, 20-Hz BLA stimulation evokes a tail of hyperpolarization following the offset of the train (Stone et al., 2011). All recorded GC neurons showed a decrease in the variability of the membrane potential following the BLA burst. When we compared the coefficient of variance (CV) for the peak of sVPMpc-PSPs and bBLA+sVPMpc-PSPs, we found a significant decrease to 75 ± 10% of baseline for 50 ms post-burst latency (Fig. 7*B*; CV, baseline sVPMpc-PSP: 0.36 ± 0.06; bBLA+sVPMpc-PSP: 0.26 ± 0.05; paired sample one-tailed *t* test, *p* = 0.03) and to 60 ± 11% of baseline CV for 250-ms latency (Fig. 7*B*; CV, baseline sVPMpc-PSP: 0.34 ± 0.07; bBLA+sVPMpc-PSP: 0.19 ± 0.05; paired sample one-tailed *t* test, *p* = 0.03). For pairings with a latency of 500 ms, no significant changes in CV were observed (Fig. 7*B*; CV, baseline sVPMpc-PSP: 0.25 ± 0.06; bBLA+sVPMpc-PSP: 0.24 ± 0.04; one-tailed *t* test, *p* = 0.40). BLA burst stimulation may serve to decrease membrane potential variability for ∼250 ms following offset of the burst, and this reduction in CV may be one mechanism by which the amygdala can modulate the gain of other circuit inputs.

In addition, a BLA burst preceding VPMpc stimulation modulated VPMpc-PSP response latencies. We pooled the data from the *n* = 8 cells for which we collected a complete dataset of post-BLA burst latencies of 50, 250, and 500 ms (*n* = 3 from the facilitating amplitude group, *n* = 5 from the suppressing amplitude group) and compared sVPMpc-PSP onset latency and time-to-peak measured at baseline (sVPMpc) and post-BLA burst (bBLA+ sVPMpc). For all neurons (*n* = 8), PSP onset latency for the bBLA+sVPMpc-PSP was significantly longer at each post-burst latency tested (Fig. 7*C*). The strongest effect was observed at a 50 ms post-burst latency where the bBLA+sVPMpc-PSP onset latency was 120 ± 3% of baseline (Fig. 7*C*; onset latency, sVPMpc-PSP: 5.8 ± 0.3 ms; bBLA+sVPMpc-PSP: 7.0 ± 0.3 ms; paired sample two-tailed *t* test, *p* = 10^−5^). At a 250 ms post-burst latency, onset latency was 110 ± 3% of baseline (Fig. 7*C*; onset latency, sVPMpc-PSP: 5.7 ± 0.3 ms; bBLA+sVPMpc-PSP: 6.2 ± 0.3 ms; paired sample two-tailed *t* test, *p* = 0.02). At a 500 ms post-burst latency, the PSP onset latency was 110 ± 2% of baseline (Fig. 7*C*; onset latency, sVPMpc-PSP: 5.7 ± 0.3 ms; bBLA+sVPMpc-PSP: 6.3 ± 0.3 ms; paired sample two-tailed *t* test, *p* = 5 × 10^−4^). Analysis of time-to-peak revealed that at a 50 ms post-BLA burst latency, the time-to-peak showed a small but significant decrease to 98 ± 3% of baseline (Fig. 7*D*; time-to-peak, sVPMpc-PSP: 17.9 ± 0.9 ms; bBLA+sVPMpc-PSP: 16.1 ± 0.6 ms; paired sample two-tailed *t* test, *p* = 0.005). Differently, for longer post-burst latencies, sVPMpc-PSP time-to-peak was increased. At 250 ms, time-to-peak was 114 ± 3% of baseline (Fig. 7*D*; time-to-peak, sVPMpc-PSP: 17.8 ± 0.9 ms; bBLA+sVPMpc-PSP: 19.6 ± 0.9 ms; paired sample two-tailed *t* test, *p* = 0.01). At 500 ms, time-to-peak was 118 ± 3% of baseline (Fig. 7*D*; time-to-peak, sVPMpc-PSP: 17.2 ± 0.9 ms; bBLA+sVPMpc-PSP: 20.0 ± 0.9 ms; paired sample two-tailed *t* test, *p* =10^−4^). When time-to-peak was measured from PSP onset rather than stimulus onset, the effect at a 50 ms post-burst latency was decreased to 87 ± 4% of baseline (Fig. 7*E*; time-to-peak from PSP onset, sVPMpc-PSP: 12.1 ± 0.7 ms; bBLA+ VPMpc-PSP: 9.1 ± 0.4 ms; paired sample two-tailed *t* test, *p* = 10^−5^). At a 250-ms delay, the time-to-peak from PSP onset was not significantly different (Fig. 7*E*; time-to-peak from PSP onset, sVPMpc-PSP: 12.5 ± 0.8 ms; bBLA+VPMpc-PSP: 13.7 ± 0.7 ms; paired sample two-tailed *t* test, *p* = 0.1). At a 500-ms post-burst delay, the time-to-peak from PSP onset was significantly increased to 122 ± 4% of baseline (Fig. 7*E*; time-to-peak from PSP onset, sVPMpc-PSP: 11.7 ± 0.7 ms; bBLA+VPMpc-PSP: 13.9 ± 0.7 ms; paired sample two-tailed *t* test, *p* = 0.001). The rise time of the bBLA+sVPMpc-PSP was significantly decreased from baseline in the 50-ms latency condition (rise time 10–90% peak amplitude, sVPMpc-PSP: 15.2 ± 3.9 ms; bBLA+sVPMpc-PSP: 7.1 ± 1.4 ms; paired sample two-tailed *t* test, *p* = 0.03; *n* = 8). This effect is consistent with the observation of opposite changes in sVPMpc-PSP onset, delayed, and time-to-peak, shortened, in the 50-ms delay condition. Altogether, these parameters highlight the complexity of the modulatory effects of 20-Hz BLA bursts preceding VPMpc stimuli at both suppressing and facilitating neurons. Nevertheless, modulation of these parameters was comparable in facilitation and suppression groups, therefore it did not explain the different effects of BLA on VPMpc-PSP amplitudes.

**Figure 8.**
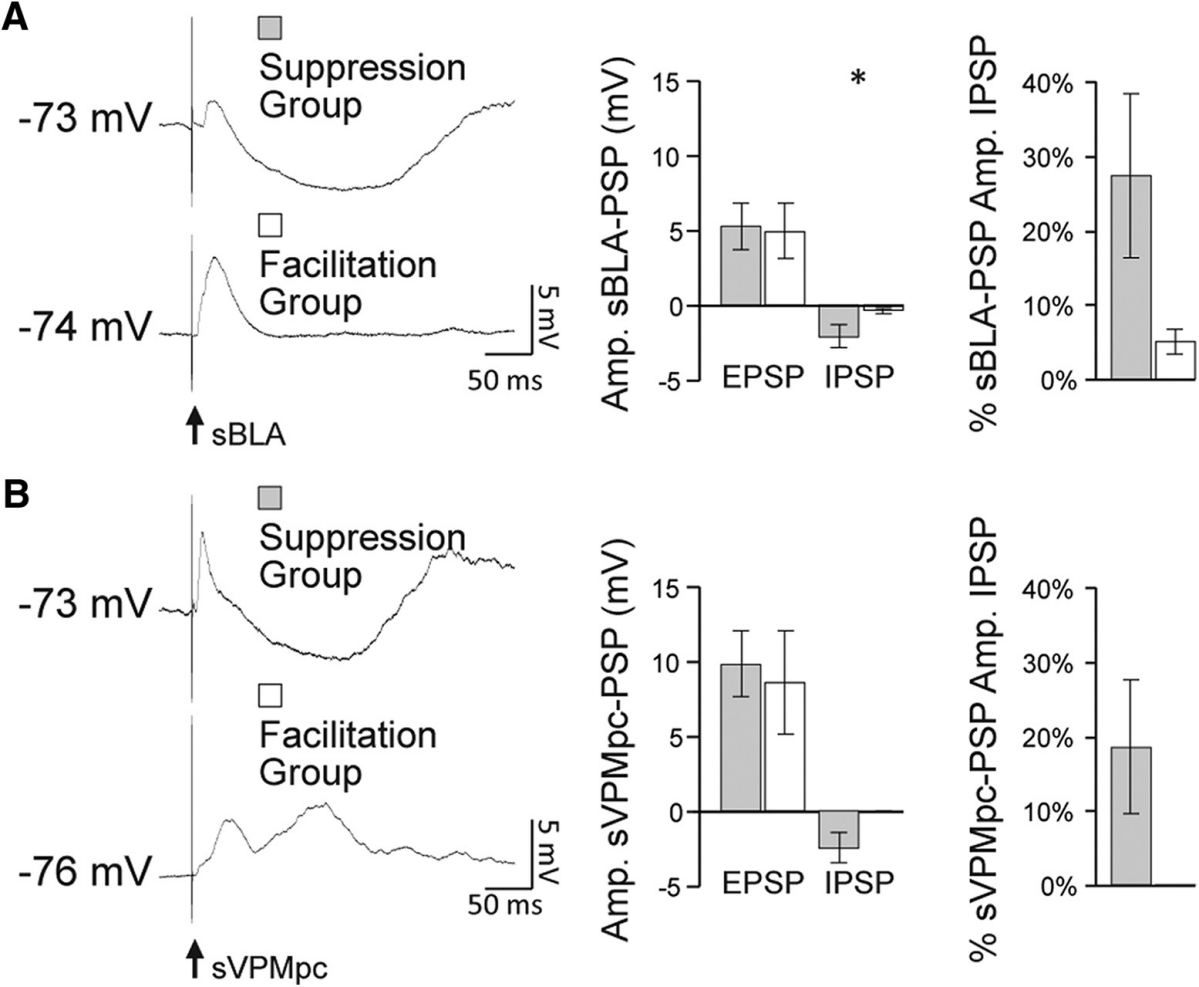
Local recruitment of inhibition determines the sign of BLA modulation of VPMpc. ***A***, left panel, Example traces for comparison of the size of the excitatory and inhibitory components of the sBLA-PSP measured at V_m_ for a cell from the suppression group (e.g., top trace) and from the facilitation group (e.g., bottom trace). Black arrow indicates BLA stimulus onset. Middle panel, Average ± SEM of the EPSP and IPSP amplitudes measured in the suppression group (gray bars) and the facilitation group (white bars) when BLA was stimulated at rest. Asterisks indicate *p* < 0.05. *y*-axis: sBLA-PSP amplitude (mV). Right panel, % contribution of the IPSP amplitude to total response height for the suppression group (gray bar) and the facilitation group (white bar). ***B***, left panel, Example traces for comparison of the size of the excitatory and inhibitory components of the sVPMpc-PSP measured at V_m_ for a cell from the suppression group (e.g., top trace) and from the facilitation group (e.g., bottom trace). These cells are the same as in ***A***. Black arrow indicates VPMpc stimulus onset. Middle panel, Average ± SEM of the EPSP and IPSP amplitudes measured in the suppression group (gray bars) and the facilitation group (white bars) when VPMpc was stimulated at rest. *y*-axis: sVPMpc-PSP amplitude (mV). Right panel, % contribution of the IPSP amplitude to total response height for the suppression group (gray bar) and the facilitation group (white bar).

We then focused on the comparison of excitatory and inhibitory components of single BLA and VPMpc evoked PSPs of the neurons receiving converging inputs. The amplitude of the hyperpolarizing component of the sBLA-PSP evoked while GC neurons were sitting at resting membrane potential was significantly larger in the group showing BLA burst-induced suppression of the sVPMpc-PSP (Fig. 8*A*, left and middle panels; peak of the BLA hyperpolarizing response, facilitation group: −0.3 ± 0.2 mV; suppression group: −2.1 ± 0.8 mV; independent sample one-tailed *t* test, *p* = 0.04). When the EPSP amplitude was added to the absolute value of the IPSP amplitude to yield a total response amplitude, the inhibitory component of the response accounted for 4 ± 2% of the BLA-evoked PSP total response amplitude for the group of neurons showing enhancement and for 25 ± 11% of the amplitude of the BLA-evoked PSP for the group of neurons showing suppression (*n* = 5; Fig. 8*A*, right panel). Figure 8*A*, left panel exemplifies this difference; the top trace is a sBLA-PSP from a cell in the suppression group showing a polysynaptic inhibitory trough at V_m_ while the bottom trace, from a cell in the facilitation group, lacks this hyperpolarization at V_m_. These data suggest that the size of the BLA-PSP inhibitory component evoked at V_m_ can influence the sign (facilitation or depression) of the modulation of a BLA burst on GC neurons’ responsiveness to VPMpc inputs. An additional difference between the two groups of neurons regards the contribution of inhibition from the VPMpc-PSP. The majority of neurons (*n* = 4/5) showing suppressed VPMpc-PSPs in response to bBLA+sVPMpc pairings responded to a single VPMpc stimulus with an excitatory response followed by an inhibitory component (Fig. 8*B*, left panel, top trace). These cells would have a mixed potential early in the sVPMpc-PSP (Fig. 3*D*). Differently, all the neurons (*n* = 3/3) showing facilitation in response to BLA-VPMpc pairings lacked a VPMpc-evoked inhibitory component (Fig. 8*B*, left panel, bottom trace). These cells have a comparatively stronger excitatory drive early in the sVPMpc-PSP (Fig. 3*E*). Taken together, the data suggest that in GC neurons with a significant inhibitory polysynaptic response to BLA stimulation at V_m_, the suppressive effect of a preceding BLA burst on the VPMpc-PSP is primarily dependent on summation of BLA-evoked inhibition and the mixed excitatory/inhibitory potential contributed by the VPMpc. In contrast, neurons displaying enhancement of the VPMpc response following a BLA burst showed no VPMpc-PSP inhibitory component and a small-to-negligible BLA-PSP inhibitory component at V_m_. In this group of neurons, the integration of thalamic and limbic inputs relied primarily on summation of the excitatory components of the PSPs, resulting in an amplification of the thalamic-evoked response amplitude. Future study is needed to determine the connectivity of the local circuit and to see whether there are any differences based on cortical layer or subdivision.

Altogether, our data show that a population of GC neurons receive convergent thalamic and limbic inputs. BLA inputs can modulate both the gain and temporal dynamics of VPMpc-evoked responses. While the temporal dynamics are similarly affected in all the GC neurons directly activated BLA and VPMpc afferents, the sign of BLA modulation of the gain of VPMpc-PSPs depends on the recruitment of local inhibition and on the relative timing of incoming thalamocortical and amygdalocortical stimuli.

## Discussion

In this paper, we present results showing that thalamic and amygdalar axonal fields are distributed in the gustatory portion of the insular cortex. While visual inspection of the data suggests that VPMpc axons are primarily distributed dorsally and BLA axons are prominently distributed ventrally in GC, imaging analysis highlighted a region of terminal field overlap. Intracellular recordings in anesthetized animals allowed us to identify a population of GC neurons receiving direct projections from both BLA and VPMpc, supporting the presence of a circuit for direct convergence and integration of stimuli carrying information about different aspects of a sensory experience.

VPMpc is considered the primary sensory thalamocortical input to GC, while BLA is thought to convey information about the affective dimensions of a taste stimulus, compatible with the interpretation that VPMpc inputs are drivers and BLA projections act as modulators (Sherman and Guillery, 1998). Consistent with this hypothesis, we report that a single preceding BLA stimulus can facilitate or depress the evoked VPMpc-PSP depending on the relative timing between stimuli. This result is related to the recruitment of excitation (early) and inhibition (late) in the BLA-PSP (Stone et al., 2011) and to the direct recruitment of feedforward inhibition by BLA (Haley et al., 2016). BLA burst stimulation further emphasizes the complex dynamics of BLA-VPMpc integration and highlights a possible role for inhibitory circuits in this process. Indeed, neurons with a substantial inhibitory component in both VPMpc-PSP and BLA-PSP at resting membrane potential showed significant suppression of VPMpc-PSPs by a preceding BLA burst. Differently, neurons whose response to either VPMpc or BLA stimulation showed a negligible inhibitory component at rest, a BLA burst facilitated the VPMpc-PSP. These results unveil a synaptic model for the generation of the rich, multidimensional taste coding performed by neurons in GC.

### Integration of sensory and limbic inputs in GC

Gustatory information reaches GC via a sensory, thalamic pathway and a limbic, amygdalar pathway. Pioneering tracing experiments showed that, in rats, taste thalamus targets the granular and dysgranular subdivisions of GC (Allen et al., 1991; Nakashima et al., 2000), while BLA targets dysgranular and agranular (Allen et al., 1991). These results have been interpreted as evidence of segregation of sensory and limbic afferents to GC, leading to the hypothesis that dysgranular and agranular GC should be considered “high order” gustatory cortices (Shi and Cassell, 1998; Sewards and Sewards, 2001). However, single unit recordings in alert animals did not support such a degree of functional segregation. Indeed, GC neurons encoding chemical identity, palatability, and responding to cross-modal stimuli that anticipate taste (Fontanini and Katz, 2006; Samuelsen et al., 2012; Gardner and Fontanini, 2014) are distributed across all divisions. Multiplexing of information could arise from polysynaptic interactions between sensory and limbic pathways, from direct, monosynaptic convergence or from a hybrid connectivity in which a group of neurons acts as direct integrations, while others are modulated via intracortical connectivity. Our data reveal an overlap of thalamic and amygdalar terminal fields in GC that extends into all three divisions of GC, albeit the density of BLA and VPMpc axons appears to show a bias similar to what was reported in previous tracing studies. Results from intracellular recordings also demonstrated functional overlap of the inputs and unveiled convergent monosynaptic responses onto a population of GC neurons distributed across all three divisions, albeit dual responders were found most frequently at depths associated with the dysgranular portion of GC. Neurons responding only to VPMpc or to BLA were also observed, supporting the possibility that the integration of these inputs follows a hybrid model consisting of a circuit of monosynaptic integrator neurons, and an additional channel that may rely on polysynaptic connectivity. As the current understanding of GC recurrent connectivity is rather limited, there is no sufficient information to make any inferences regarding polysynaptic integration. The presence of a distributed population of neurons that directly receives VPMpc and BLA afferents, however, suggests that in addition to neurons receiving inputs solely from one source and potentially specializing in either sensory or affective processing, a significant proportion of GC neurons can engage in the integration of sensation and emotion.

Our study is not meant to be an exhaustive analysis of the synaptic connectivity of BLA and VPMpc inputs to GC. Given the challenging nature of intracellular recordings *in vivo*, a different experimental approach may be more appropriate for fine scale analysis of connectivity. However, in our experience, neurons receiving convergent inputs were found often, suggesting that the functional overlap of thalamic and limbic afferents is more prominent than previously anticipated. The primary limiting factor to an extensive analysis of connectivity in our study was not the identification of neurons receiving direct BLA and VPMpc input, but the high demands on the duration and stability of intracellular recordings.

### Temporal dynamics of the thalamocortical synapse

When VPMpc is electrically stimulated, GC pyramidal cells respond with a postsynaptic depolarization with delays and a reversal potential consistent with monosynaptic, glutamatergic excitation. Following the EPSP, 50% of the cells showed a polysynaptic, GABAergic inhibitory component. Whether this IPSP is generated via feedforward inhibition, as is the case with the projection of BLA inputs to GC (Haley et al., 2016), or via a feedback mechanism is not yet known. The other 50% of GC neurons lacked an inhibitory component and responded with a multi-synaptic depolarization following the initial EPSP, suggesting recruitment of recurrent excitatory connectivity. The response heterogeneity suggests that VPMpc inputs can preferentially recruit excitation and inhibition depending on target GC neurons and their local connectivity. Whether the cell’s response profile depends on its location in the cortical layers and/or subdivisions deserves future investigation.

### Effects of amygdalar inputs on processing of thalamic inputs

The amygdala exerts a strong modulatory effect on GC. Studies in anesthetized rodents demonstrate that stimulation of the amygdala can increase or decrease firing rates in GC neurons (Yamamoto et al., 1984a; Hanamori, 2009). These effects may relate to time varying synaptic potentials evoked by amygdalar stimulation (Stone et al., 2011). In GC, excitation by BLA likely relies on the organization of the BLA-GC circuit, in which excitation is likely mediated by monosynaptic glutamatergic amygdalar inputs onto both pyramidal and inhibitory neurons (Haley et al., 2016), and to the suppression of firing results from di-synaptic or multi-synaptic inhibition. Laminar differences in the synaptic organization of amygdalar afferents in GC (Haley et al., 2016) are likely to add further complexity to the effects of BLA stimulation.

Recent studies in alert animals reported that inactivating BLA can substantially affect taste processing and responses to anticipatory cues in GC (Piette et al., 2012; Samuelsen et al., 2012). BLA inactivation does not fully eliminate a taste-evoked response, pointing to a modulatory role for BLA inputs onto GC neurons. Differently, inactivation of VPMpc abolishes taste responses (Samuelsen et al., 2013), consistent with the idea that VPMpc inputs act as drivers (Sherman and Guillery, 1998). An additional effect reported in BLA inactivation studies in awake animals is that the firing rates of GC neurons are changed bidirectionally, an effect consistent with the mixed excitatory/inhibitory action of BLA on GC and its time varying nature. The results presented here, further elucidate the synaptic basis of this effect. Finally, the dynamics of BLA modulation of VPMpc responses are time-dependent and activity-dependent with distinct effects depending on whether VPMpc-PSPs were preceded by a single BLA stimulus or a train of BLA stimuli. The modulation of GC neurons’ responsiveness to VPMpc stimulation by a single preceding BLA stimulus depends strongly on the temporal dynamics of excitation and inhibition of the evoked BLA-PSP. The integration of BLA and VPMpc inputs becomes more complex if a BLA burst precedes a VPMpc stimulus. GC neurons responding to single BLA and VPMpc stimuli with time varying PSPs in which an early, depolarizing, excitatory component was followed by a hyperpolarizing, inhibitory component showed significant suppression of VPMpc responses following the BLA burst, showing suppression or facilitation depending on the magnitude of the polysynaptic inhibitory response evoked by VPMpc and BLA stimuli. It is important to note that 100% of the neurons we tested in these protocols belonged to either the suppression or facilitation group; none of the recorded neurons showed absence of BLA modulation of VPMpc responses. Our experiments do not identify if neurons with stronger BLA-evoked inhibition differ in their patterns of local and long-distance connectivity. Future studies will combine tracing methods and precisely targeted recordings to identify additional sources of response differences.

### Significance for palatability processing in GC

Pharmacological, lesion, and electrophysiological experiments in alert rodents provide evidence of BLA’s role in processing hedonic information in GC (Schafe et al., 1998; Escobar and Bermúdez-Rattoni, 2000; Escobar et al., 2002; Ferreira et al., 2005; Reilly and Bornovalova, 2005; St Andre and Reilly, 2007; Grossman et al., 2008; Fontanini et al., 2009; Piette et al., 2012; Samuelsen et al., 2012; Molero-Chamizo and Rivera-Urbina, 2017). Pharmacological silencing of BLA affects taste-evoked firing rates of GC neurons (Piette et al., 2012; Samuelsen et al., 2012). Most neurons reduced their firing activity, but a significant portion also increased or showed bimodal modulations. The heterogeneity of this effect is consistent with our results, indicating that the effect of BLA on firing likely depends on the amount of inhibition recruited by incoming stimuli and by the relative timing of VPMpc and BLA evoked activity.

The complex effects of BLA inactivation on firing rates resulted in a dramatic reduction of palatability coding, with marginal effects on chemosensory coding (Piette et al., 2012), supporting a modulatory role of BLA activity on taste responses. In addition, the role of BLA in GC palatability coding extends to learning. Indeed, changes in palatability rapidly affect firing rates in BLA neurons, while their effects on taste responses in GC have relatively small effects on the early phase of the taste-evoked response, but significantly modulate the late phase of a taste response (Grossman et al., 2008; Piette et al., 2012). Modulation of taste evoked activity by BLA may thus facilitate a shift in palatability coding. While the timing of the effects we report may appear, at first glance, not necessarily compatible with the timing of the effects on palatability coding observed in awake animals, it is important to keep in mind that our current study focuses on subthreshold synaptic activity, while previous work quantified firing rates. In addition, the state of the cortical circuit, and thus the time window for integration in anesthetized animals in this study may differ from that affecting integration in awake subjects. While our results do not directly address state changes nor plasticity of the BLA-GC synapse, they suggest that the differential recruitment of inhibition may provide a mechanism to selectively gate plasticity in specific elements of the circuit. Future experiments will directly address this important issue.

## Synthesis

Reviewing Editor: Christian Hansel, University of Chicago Decisions are customarily a result of the Reviewing Editor and the peer reviewers coming together and discussing their recommendations until a consensus is reached. When revisions are invited, a fact-based synthesis statement explaining their decision and outlining what is needed to prepare a revision will be listed below. The following reviewer(s) agreed to reveal their identity: Donald Katz.

The comments of two referees are summarized / synthesized below.

This new manuscript, which continues the work of the premier taste electrophysiology lab connecting action potential coding in awake rats to synaptic and circuit function, involves quietly heroic in vivo intracellular recordings of GC pyramidal cells, with the purpose being a characterization of the responses of these cells to thalamic input, amygdalar input, and a combination of inputs. This ephys work is supported by an anatomy experiment in which terminal fields of those two input pathways are mapped simultaneously. The authors report substantial overlap of these fields, and go on to show substantial convergence of the two inputs on single GC neurons; in this population of neurons receiving monosynaptic input from both pathways, amygdalar input proves to modulate cortical responses to thalamic input, in a dynamic manner that is dependent (in a way that helps explain differences in the responses of individual neurons) on amount of polysynaptic inhibition.

This work advances the field by demonstrating that thalamic and limbic inputs to a primary sensory cortex, in this case the gustatory cortex (GC), converge and interact in time-dependent ways. This supports the idea that cells in the GC integrate more information than just the physiochemical attributes of a taste stimulus. Furthermore, the manuscript provides evidence that the limbic input from the basolateral amygdala may modulate the GC by controlling the excitatory/inhibitory balance. That said, the functional implications of this aspect of limbic modulation are unknown. Furthermore, the small number of cells recorded in this study seriously limits the interpretation of the results, in my opinion.

  This work, while not flashy (electrical stimulation...how classic!), is both highly impressive and highly important. At the most basic level, it casts serious doubt on the assertion, made based on fairly old anatomical work, that different parts of GC are segregated for either sensory (thalamic input) or “secondary” (amygdalar input) functions; the simultaneous tracing shows overlap between inputs to be far more substantial than previously thought, and the electrophysiology shows even more extensive convergence. More specifically, this work represents another nail in the coffin of the errant (if high-profile) suggestion that taste input is processed by small, circumscribed sub-regions of GC. Finally, the work on the dynamics of convergence provides further scaffolding for a model with the potential to explain how GC turns its input into coding of both taste and palatability. The data appear to be of very high-quality-while the N’s are (understandably) small, the effects are robust and reasonable (notable example: while few neurons were held through a full set of both thalamic and amygdalar stimuli, ALL of them fell cleanly into one of two functional groups, and NEITHER of those groups were “no effect”)-and the control analyses clever. I have only a few requests and suggestions that I’d like handled prior to acceptance, and these should be easy for the authors to handle.   Most centrally, I’d like to see the authors do a more thorough consideration of possible “models” for GC organization (for instance, on lines 68-74 of the Introduction, they seem to suggest only 2, without noting the possibility of a 3rd “hybrid” model). More specifically, I’d like to see some Bayesian-esque analysis of the likelihood of input convergence given the likelihood of neurons responding to each input individually-is it random, or is convergence more (or less) likely than one would expect from chance? I know that samples are somewhat small for such analyses, but they would be really useful; while I fully support the authors’ refusal to succumb to simplistic “the fact that some neurons take input from both, and others from only one, suggests the existence of multiple, separately functional networks” thinking, it’s worth asking if some structural organization is at play here.   I appreciate the gargantuan effort required to collect each individual data point, but it’s hard not to wish that the data for Figure 5D (for instance-I feel similarly about later figures) was binned smaller than “12-51 msec.” Is there any way that the authors could provide a more fine-grained analysis of the match between basic time courses of PSPs and time courses of amygdalar modulation of thalamic PSPs? Relatedly, the authors should be careful about how they interpret the differences between distributions (e.g., Figure 7A) in the absence of direct statistical comparisons of the distributions.   Finally, with regard to the last bit of the Results, the authors’ conclusion is simple and compelling. Still, it would be great to see (if dataset size allows) a little deeper dive. Clearly, the lack of inhibitory components of PSPs predicts one result, and the presence of polysynaptic inhibition predicts another. Is that effect linear, however? Is it ever the case that one input to a GC neuron includes inhibition while the other doesn’t, and if so which seems “more important” for the result?   (Even more) trivial concerns.   There’s no need to start the Methods section with a sentence assuring the reader that the methods used “are described...below.”   Given the recent push at the NIH (funder of this work) for sex to be explicitly considered as a biological variable, it would be a good idea to motivate the use of only female rats.   An explicit statement of the lack of fluorescence of GC cell bodies would enhance confidence in the authors’ suggestion that AAV only reveals anterograde processes.   Why was the yield of biocytin staining so low?   A good portion of the first part of the Results section seems archival and lacking in direction (lines 444-475, for instance). The authors should probably both clarify how the information described relates to the hypotheses tested in the paper and condense this material into a more brief, easily-digestible form.   The statement “time-varying temporal dynamics (line 566) is a little like the song title “The Big Rio Grande River"-aggressively redundant. Probably any one of the 3 words will serve.

This manuscript is a study of the convergence and modulation of inputs to the gustatory cortex (GC) originating from the basolateral amygdala (BLA) or the parvicellular portion of the ventroposteromedial thalamus (VPMpc) in anesthetized rats in vivo. Result show that input from the BL converge on the same cells a those that receive VPMpc input in the GC and that its function is to modulate gain. The general conclusion is that the BLA modulates excitatory and inhibitory balance in the GC circuit. The experiments are well designed and expertly conducted. Data are clear and intriguing. The authors might consider the following points:   Further MAJOR COMMENTS   Lines 401-405. Given what is shown in Fig. 1, these statements are an exaggeration. From my view, there is minimal overlap in these two regions. However, I agree that the dysgranular GC does show the most extensive VPMpc/BLA overlap, albeit limited.   There seem to be relatively few cells in each of the various categories described in this manuscript. I understand that the population of cell types is heterogeneous, but when the various effects were categorized, very few cells populated each class. This situation runs the risk of the study being underpowered...that is, a handful of cells may be too few to make the claims that are made about significance if the findings for the circuit dynamics in the GC.   Lines 875-876. There is no indication of how rare or common these VPMpc/BLA convergent cells are. Were they hard to find? How many cells were tested before one of these could be identified?   There is only a cursory mention of the potential role of the location of cells in various layers of GC.   The Discussion is very long and could be shortened substantially. In my opinion, the authors expand the interpretation of their findings, which are relatively modest, but genuinely intriguing, well beyond what they found. In contrast, there is little discussion of the potential limitations of their findings.   MINOR COMMENTS   Lines 86-88. These assertions need supporting references.   Lines 377-383. Text describing anatomical methods is repeated in the Results section. This occurs several times in Results   Lines 452-454. Were there any animals in which the placement of the VPMpc stimulating electrode was not verified?
